# Multigram-scale flow synthesis of the chiral key intermediate of (–)-paroxetine enabled by solvent-free heterogeneous organocatalysis†
†Electronic supplementary information (ESI) available: Synthetic procedures, additional batch and flow reaction data, characterization data, copies of NMR spectra and HPLC chromatograms. See DOI: 10.1039/c9sc04752b


**DOI:** 10.1039/c9sc04752b

**Published:** 2019-10-18

**Authors:** Sándor B. Ötvös, Miquel A. Pericàs, C. Oliver Kappe

**Affiliations:** a Institute of Chemistry , University of Graz , NAWI Graz , Heinrichstrasse 28 , A-8010 Graz , Austria . Email: sandor.oetvoes@uni-graz.at; b Institute of Chemical Research of Catalonia (ICIQ) , The Barcelona Institute of Science and Technology (BIST) , Av. Països Catalans 16 , E-43007 Tarragona , Spain; c Departament de Química Inorgànica i Orgànica , Universitat de Barcelona (UB) , E-08028 Barcelona , Spain; d Center for Continuous Synthesis and Processing (CCFLOW) , Research Center Pharmaceutical Engineering (RCPE) , Inffeldgasse 13 , A-8010 Graz , Austria . Email: oliver.kappe@uni-graz.at

## Abstract

The catalytic enantioselective synthesis of the chiral key intermediate of the antidepressant (–)-paroxetine is demonstrated as a continuous flow process on multi-gram scale. The critical step is a solvent-free organocatalytic conjugate addition followed by a telescoped reductive amination–lactamization–amide/ester reduction sequence. Due to the efficient heterogeneous catalysts and the solvent-free or highly concentrated conditions applied, the flow method offers key advances in terms of productivity and sustainability compared to earlier batch approaches.

## Abstract 2

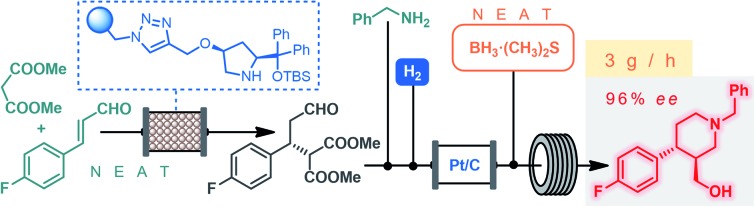
The continuous flow synthesis of the chiral key intermediate of (–)-paroxetine is demonstrated *via* a solvent-free organocatalytic conjugate addition followed by a telescoped reductive amination–lactamization–amide/ester reduction sequence.

## 

(–)-Paroxetine is a selective serotonin reuptake inhibitor marketed as Paxil/Seroxat which is widely used for the treatment of depression, anxiety and panic disorder.^1^ The published procedures for its synthesis involve 10–15 reaction steps and typically utilize chiral auxiliaries, classical resolution methods, enzymatic asymmetrizations or naturally occurring homochiral starting materials to introduce asymmetry.^2^ In most of these synthetic routes, the corresponding ((3*S*,4*R*)-4-(4-fluorophenyl)piperidin-3-yl)methanol (**1**) is obtained as key chiral intermediate which can readily be converted into the active pharmaceutical ingredient (API) upon etherification with sesamol and removal of the protecting group on nitrogen (Fig. 1).^2^ Catalytic enantioselective transformations have also been exploited for the synthesis of (–)-paroxetine or its advanced intermediates.^3^ These methods require less synthetic steps and provide more direct access to the target API, but their applicability for manufacturing is limited by the low productivity of the catalytic asymmetric key step.

**Fig. 1:**
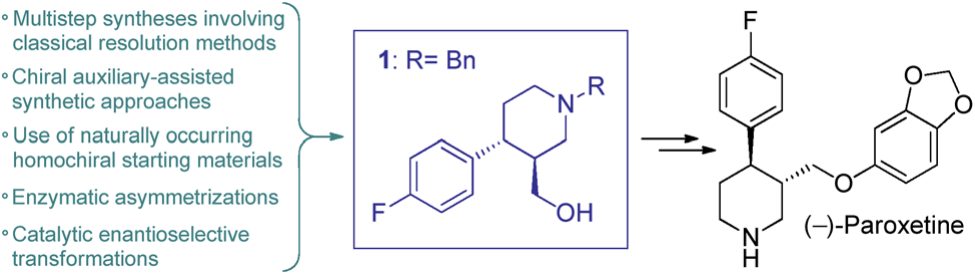
Antidepressant (–)-paroxetine and its chiral key intermediate.

Continuous flow processing offers numerous benefits over conventional batch syntheses.^4^ From the viewpoint of pharmaceutical manufacturing, the safe and scalable access to hazardous chemistries^5^ and the ability to combine multistep reactions into telescoped flow sequences is particularly appealing.^6^ Accordingly, there is an increasing number of reports on multistep flow syntheses of APIs and pharmaceutically relevant intermediates.^6^,^7^ However, these studies rarely involve catalytic enantioselective transformations.^8^ The overwhelming majority of the examples reported to date cover achiral products, or, in the case of chiral targets, asymmetry is typically derived from commercially available chiral building blocks without exploiting enantioselective transformations.^6^,^7^

Inspired by the above findings and limitations, we aimed for a continuous flow strategy for the catalytic enantioselective synthesis of the chiral key intermediate of (–)-paroxetine. We intended to use only inexpensive achiral starting materials in combination with a highly efficient organocatalytic approach. The synthetic strategy relies on the enantioselective asymmetric conjugate addition between 4-fluorocinnamaldehyde and dimethyl malonate to yield chiral aldehyde **2** which can subsequently be converted into phenylpiperidinone **3***via* a three-step cascade involving imine formation, reduction and lactamization, to finally provide key intermediate **1** by means of reduction of the amide and ester moieties (Scheme 1).

**Scheme 1:**
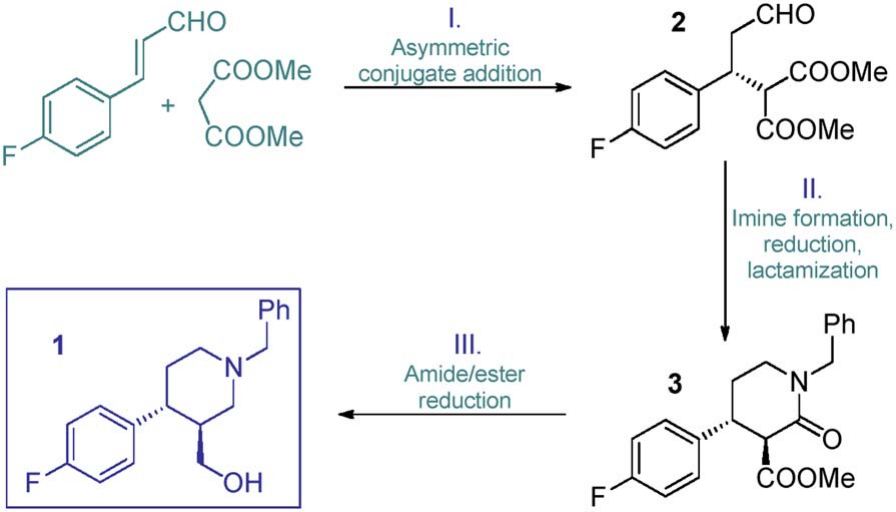
Synthetic strategy toward **1**.

While most of the published target-oriented organocatalytic asymmetric syntheses involve homogeneous catalysts under batch conditions,^9^ we aimed to utilize a resin-bound organocatalyst under flow conditions.^10^ This not only enables facile product isolation, but, if catalyst deactivation can be minimized, readily facilitates scale-out of the flow synthesis by increasing processing time.^11^

We selected a polystyrene-supported *cis*-4-hydroxydiphenylprolinol TBS ether (**4**, Table 1) as catalyst, which was recently developed as a modified version of classical *trans* analogues,^12^ and was employed for additions of hydroxylamine derivatives to enals.^13^ This catalyst has not yet been investigated in conjugate additions of malonates, however, becouse of the *cis* arrangement and the TBS protecting group, we anticipated higher activity and improved robustness than in earlier cases.12*b*

**Table 1:** Optimization of the organocatalytic conjugate addition under solvent-free flow conditions^*a*^

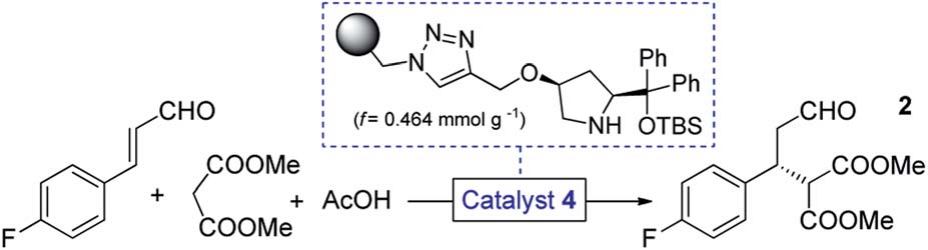					
#^*b*^	Malonate (equiv.)	Flow rate (μL min^-1^)	*T* (°C)	Conv.^*c*^ (%)	ee^*d*^ (%)
1	9	100^*e*^	50	91	98
2	3	100^*e*^	50	81	98
3	3	100^*e*^	60	89	97
4	3	100^*e*^	70	94	95
5	2	70^*f*^	60	93	97
6	2	50^*g*^	60	94	97
7	2	20^*h*^	60	99	95

^*a*^1 equiv. 4-fluorocinnamaldehyde, 0.6 equiv. AcOH as additive, 1 g catalyst **4** in Omnifit® column, solvent-free.

^*b*^No side product formation, chemoselectivity was 100% in all reactions.

^*c*^Determined by ^1^H-NMR analysis of the crude product.

^*d*^Determined by chiral HPLC.

^*e*^ *t*_*r*_= 14 min.

^*f*^ *t*_*r*_= 20 min.

^*g*^ *t*_*r*_= 28 min.

^*h*^ *t*_*r*_= 70 min.

Initial trials with catalyst **4** (*f* = 0.464 mmol g^–1^) in the 4-fluorocinnamaldehyde-dimethyl malonate conjugate addition were carried out as small-scale batch reactions. To our delight, aldehyde **2** (*cf.*Scheme 1) was formed with up to 98% ee under an array of reaction conditions (Tables S1–S3†). It was confirmed that the reaction tolerated different solvents and conversion was improved in the presence of AcOH as additive. After the successful preliminary experiments, 1 g of catalyst **4** was placed in an Omnifit® glass column (10 or 6.6 mm ID, adjustable height) and the reaction was investigated under continuous flow conditions. Initially, CH_2_Cl_2_ was employed as solvent as it provided the best results in terms of ee and conversion during the batch experiments, and at the same time it ensured good swelling of the polystyrene-based catalyst carrier. It was found that the rate of reaction was significantly enhanced without loss of ee upon heating the catalyst bed to 50 °C (Table S5†). It was also observed that an increase in reactant concentration resulted in a significant improvement in conversion (Table S6†). This important finding inspired us to investigate the reaction further under solvent-free conditions.

Eliminating the solvent from a synthetic transformation offers significant advantages in terms of process costs, sustainability and productivity.^14^ Notably, there are only very few examples for solvent-free reactions using solid-supported organocatalysts,^15^ and, to the best of our knowledge, not involving continuous flow techniques. Gratifyingly, catalyst **4** performed well under neat conditions, despite the lower swelling of the carrier resin and thus the smaller bed volume in the more polar medium (Fig. S1†). During systematic optimization of the reaction conditions, we managed to reduce the malonate excess to 2 equiv. by using a residence time of 20 min (70 μL min^–1^ flow rate) at 60 °C, and reached 93% conversion with complete chemoselectivity and 97% ee (Tables 1 and S7–S9†). In this optimum reaction mixture, the concentration of 4-fluorocinnamaldehyde was 2.48 M as determined experimentally. To evaluate the preparative capabilities per one lab day, the above conditions were directly utilized for scale-out in a 7 h long continuous flow experiment (Fig. 2). 17.26 g of chiral aldehyde **2** were isolated after simply removing unreacted components by evaporation (84% isolated yield, 97% ee). The large-scale synthesis offered a productivity of 2.47 g h^–1^ of pure product, which is outstanding among continuous flow organocatalytic processes reported to date.^10^ The scale-out study was completed using only 1 g (0.464 mmol) of catalyst **4**. Despite the neat conditions, this proved highly robust with practically constant selectivity (95–98% ee and 100% chemoselectivity) and only a small decrease in catalytic activity (93–85% conversion; see Fig. S2† for details). The above data result in an effective catalyst loading of 0.6 mol% and an accumulated TON of 132 for the experiment. The space-time yield (STY) for this reaction was calculated as 1.76 kg L^–1^ h^–1^. Notably, the process involved low amount of waste formation as indicated by an E-factor of only 0.7. Notably, after the first 7 h long experiment, the same batch of organocatalyst was reused in two more preparative-scale runs to accumulate **2** for the optimization of the next step. (See details in the ESI†).

**Fig. 2:**
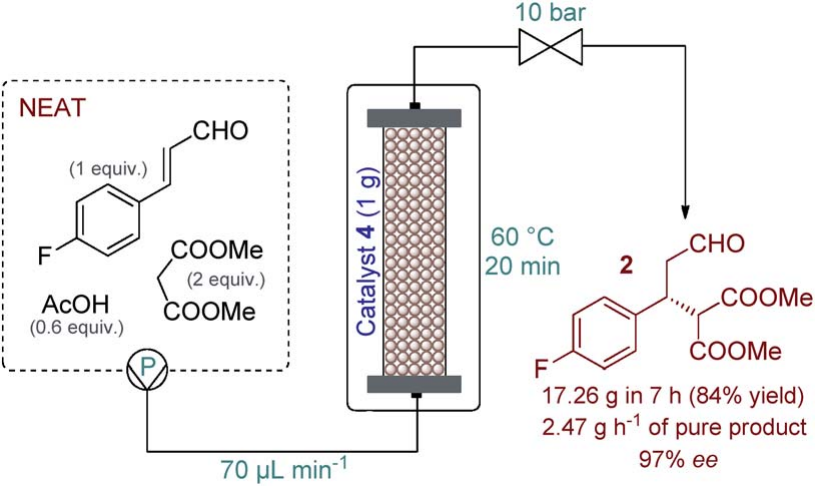
Multigram-scale continuous flow organocatalytic synthesis of chiral aldehyde **2**.

After having established a reliable flow process for the synthesis of chiral aldehyde **2**, we next turned our attention to the subsequent tandem reductive amination–lactamization to yield lactam **3** (*cf.*Scheme 1), which was previously achieved exclusively in batch, typically in the presence of NaBH(OAc)_3_.^3^ Taking environmental aspects into consideration, we exploited instead a heterogeneous catalytic approach using 5% Pt/C as catalyst and H_2_ gas.^16^ A stainless steel column (4.6 mm ID, 100 mm height) was used as catalyst bed and was charged with a mixture of 200 mg of 5% Pt/C and 400 mg of activated charcoal. H_2_ gas was introduced into the system from a gas cylinder using a mass flow controller (MFC). It was observed that the reductive amination readily took place with benzylamine as reaction partner, but in the case of lower temperatures or an excess of **2**, significant amount of double alkylation occurred, indicating incomplete lactamization (Tables 2, S11 and S13; Fig. S4†). This could easily be remedied by pumping the starting materials in a 1 : 1 ratio and heating the reactor to 100 °C. Gratifyingly, the ee was around 96% in all the experiments, and the desired *trans* lactam **3** was formed as the major product. Interestingly, the diasteromeric ratio was strongly dependent on the temperature applied: it reached a maximum of 93 : 7 (*trans*/*cis*) at 100 °C (Tables 2 and S11†). In order to maximize productivity, the effects of the reactant concentration were also explored (Tables 2 and S13†). We were delighted to find that the reaction proceeded smoothly close to the solubility limit at concentrations as high as 2.0 M in 2-MeTHF, which was chosen as a greener alternative to traditional solvents.^17^ Utilizing the optimum conditions as shown in Table 2, entry 7, the system proved stable during multiple preparative-scale runs, and resulted in *ca.* 4 g h^–1^ of pure product, with isolated yields of ≥96% and ee of 96%. Importantly, the catalytic process was very clean: according to ICP MS measurements, practically no metal leaching occurred from the catalyst bed, and inorganic byproducts were not formed.

**Table 2:** Synthesis of lactam **3**: effects of reaction conditions on the tandem reductive amination–lactamization sequence

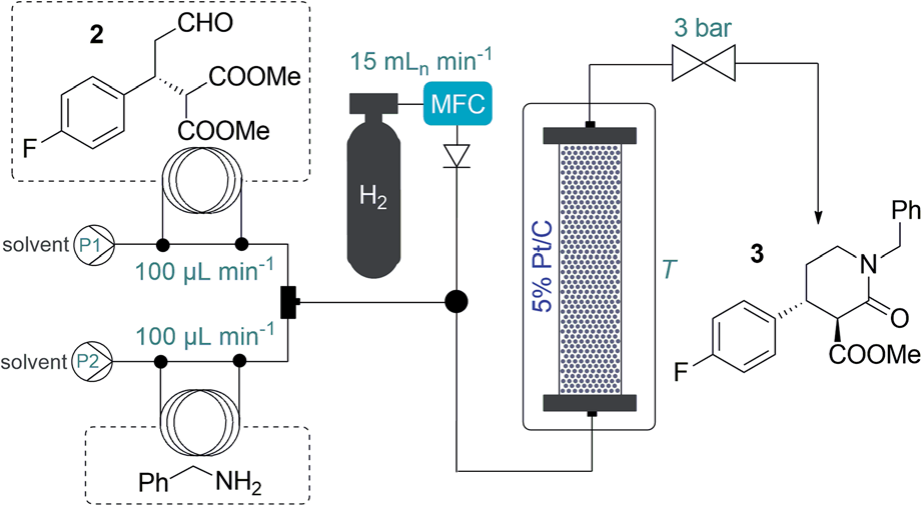						
#^*a*^	*c* (M)		*T* (°C)	Conv.^*c*^ (%)	Chemosel.^*c*^ (%)	*trans*/*cis*^*c*^
	**2**	BA^*b*^				
1^*d*^	0.2	0.2	25	95	42^*f*^	56 : 44
2^*d*^	0.2	0.2	50	98	94^*g*^	78 : 22
3^*d*^	0.2	0.2	80	100	100	89 : 11
4^*d*^	0.2	0.2	100	100	100	93 : 7
5^*d*^	0.26	0.2	100	100	70^*h*^	91 : 9
6^*e*^	1.0	1.0	100	100	100	93 : 7
7^*e*^	2.0	2.0	100	100	100	93 : 7

^*a*^95–96% ee was measured in all reactions (by using chiral HPLC).

^*b*^BA stands for benzylamine.

^*c*^Determined by ^1^H-NMR analysis of the crude product.

^*d*^Toluene as solvent.

^*e*^2-MeTHF as solvent.

^*f*^58% dialkylation.

^*g*^6% dialkylation.

^*h*^30% dialkylation.

Because of the need for extreme conditions and/or specialist catalysts,^18^ a heterogeneous catalytic hydrogenation approach would not have been feasible for the subsequent amide/ester reduction at lactam **3**. In search for an appropriate method to yield key intermediate **1**, we did not consider typical LiAlH_4_ reductions due to the formation of large amounts of metallic waste and the possible incompatibility with larger-scale flow operations (*e.g.* precipitation).^2^ Instead, we focused on borane reductions, a well-established technology frequently implemented in the pharmaceutical industry on scale.^19^ As a result of the risks associated (thermal decomposition or formation of B_2_H_6_ and H_2_ in the presence of moisture), borane complexes are typically employed as 1–2 M solutions either in batch or in flow.^19^,^20^ After promising preliminary experiments with BH_3_·THF and BH_3_·dimethylsulfide (DMS) complex solutions (Tables S14 and S15†), we attempted the amide/ester reduction by using neat BH_3_·DMS (10 M) as reducing agent with the aim to maximize productivity. To this end, a 1.0 M solution of **3** in dry 2-MeTHF and the reducing agent were pumped as separate feeds, and the combined mixture was directed through a reaction coil (PFA tubing, 1/8′′ OD, 1.58 mm ID) heated to 90 °C. The effects of individual flow rates were carefully examined (Table 3) to find out that a residence time of 30 min in combination with a substrate/reducing agent ratio of around 1 : 5 is optimal to quantitatively and selectively reduce both ester and amide moieties. Importantly, the direct application of the neat complex was safe under the strictly controlled flow conditions, and it ensured a productivity of 3.94 g h^–1^ of pure product, which is unprecedented among continuous borane reductions using solutions of the reducing agent.^20^ The enantiomeric purity was not affected by the reaction (96% ee).

**Table 3:** Completing the flow synthesis of **1**: Optimization of the BH_3_·DMS-mediated amide/ester reduction

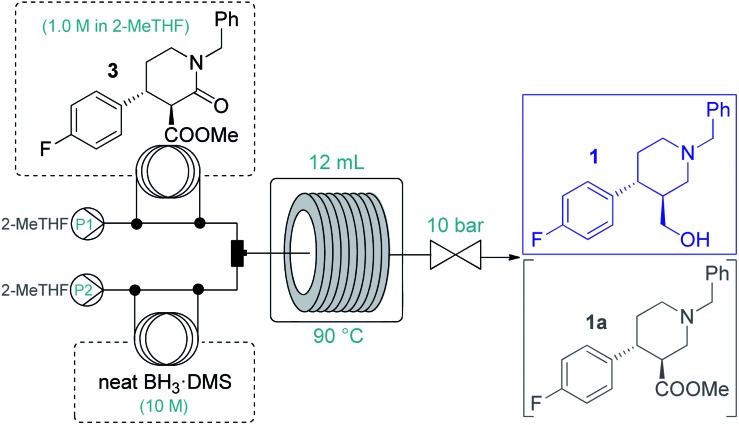					
#^*a*^	Flow rate (μL min^–1^)		**3**/BH_3_·DMS ratio	Conv.^*b*^ (%)	Chemosel.^*b*^ ^,^^*c*^ (%)
	P1	P2			
1^*d*^	200	200^*e*^	1 : 10	96	74
2	100	100^*f*^	1 : 10	100	100
3	130	70^*f*^	1 : 5.4	100	100
4	140	60^*f*^	1 : 4.3	100	95
5	150	50^*f*^	1 : 3.3	99	76
6	260	140^*e*^	1 : 5.4	100	100
7	390	210^*g*^	1 : 5.4	100	93

^*a*^95-96% *ee* was measured in all reactions (by using chiral HPLC).

^*b*^Determined by ^1^H-NMR analysis of the crude product.

^*c*^ **1a** formed as minor product in entries 1, 4, 5 and 7.

^*d*^At 50 °C.

^*e*^ *t*_*r*_= 30 min.

^*f*^ *t*_*r*_= 60 min.

^*g*^ *t*_*r*_= 20 min.

After successful step-by-step optimization, we finally wanted to combine the catalytic reductive amination–lactamization and the subsequent amide/ester reduction into an uninterrupted flow sequence (Fig. 3, S5 and S6†).^21^ Both individual steps were achieved in 2-MeTHF, therefore no solvent switch was necessary in the telescoped synthesis. Similar to the stepwise process, 2.0 M solutions of aldehyde **2** and benzylamine were pumped as separate feeds (P1 and P2, each at 100 μL min^–1^) and were combined with H_2_ gas before passing through a catalyst bed packed with a mixture of 200 mg of 5% Pt/C and 400 mg of activated charcoal. During reductive amination, one equivalent of water is released which must be removed in order to prevent decomposition of BH_3_·DMS downstream. The gas–liquid mixture exiting the Pt/C column was therefore directed through a cartridge packed with 5 g of freshly activated 4 Å MS. Excess H_2_ was separated continuously from the liquid mixture through a buffer flask. The dried and degassed feed containing a *ca.* 1 M solution of lactam **3** was re-incorporated through a 3-port valve at 200 μL min^–1^ (P3) and was combined with a stream of neat BH_3_·DMS (P4, at 110 μL min^–1^) for amide/ester reduction during passage through a reaction coil (PFA tubing, 1/8′′ OD, 1.58 mm ID) at 90 °C. The telescoped system proved stable, and the eluting product solution was collected continuously for 100 min after reaching steady state. After work-up and chromatographic purification, 4.95 g of analytically pure phenylpiperidine **1** was isolated (83% yield) with an excellent ee of 96%. The STY of the process was calculated as 0.31 kg L^–1^ h^–1^ and productivity was 2.97 g h^–1^ of pure product, which is a significant improvement compared to previously described approaches towards this class of target molecules.^3^

**Fig. 3:**
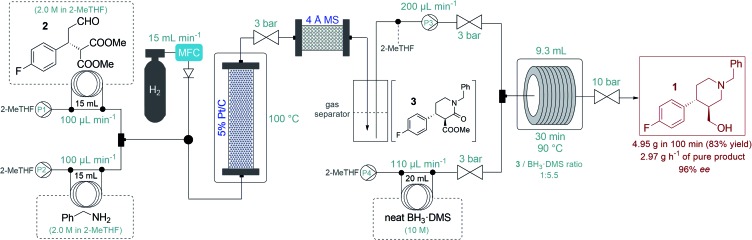
Flow synthesis of phenylpiperidin **1***via* telescoped reductive amination–lactamization–amide/ester reduction sequence.

## Conclusions

For the first time, a continuous flow process was developed for the asymmetric synthesis of phenylpiperidine **1**, the chiral key intermediate of (–)-paroxetine. The critical step of the process was a solvent-free enantioselective conjugate addition in the presence of a polystyrene-supported *cis*-4-hydroxydiphenylprolinol as heterogeneous organocatalyst. The chiral adduct was processed further *via* a telescoped reductive amination–lactamization–amide/ester reduction sequence, which took advantage of a heterogeneous catalytic hydrogenation approach and the application of neat BH_3_·DMS as reducing agent unprecedented in earlier flow reactions. The solvent-free (or highly concentrated) conditions in combination with the remarkably robust catalysts enabled a significant chemical intensification, leading to a productivity of **1** on multigram per hour scale. In addition, the process offered high chemo- and stereoselectivity generating minimal amounts of waste, demonstrated by a cumulative E-factor of 6.^22^ The only solvent used in the synthesis is 2-MeTHF, a bio-derived solvent.

## Conflicts of interest

There are no conflicts to declare.

## Supplementary Material

Supplementary information
