# Supplementary material for: Multigram-scale flow synthesis of the chiral key intermediate of (–)-paroxetine enabled by solvent-free heterogeneous organocatalysis
Source: Chem Sci. 2019 Oct 18;10(48):11141–6. doi: 10.1039/c9sc04752b (PMC7069365; doi:10.1039/c9sc04752b)
Supplement: Supplementary file 1 [file SC-010-C9SC04752B-s001.pdf]

Electronic Supplementary Material (ESI) for Chemical Science.
This journal is © The Royal Society of Chemistry 2019

SUPPORTING INFORMATION

for

Multigram-Scale Flow Synthesis of the Chiral Key Intermediate of (–)-Paroxetine Enabled by Solvent-Free Heterogeneous Organocatalysis

Sándor B. Ötvös,*[,a] Miquel A. Pericàs,[b,c] and C. Oliver Kappe*[,a,d]

[a]Institute of Chemistry, University of Graz, NAWI Graz, Heinrichstrasse 28, A-8010 Graz, Austria

[b]Institute of Chemical Research of Catalonia (ICIQ), The Barcelona Institute of Science and Technology (BIST), Av. Països Catalans 16, E-43007 Tarragona, Spain

[c]Departament de Química Inorgànica i Orgànica, Universitat de Barcelona (UB), E-08028 Barcelona, Spain

[d]Center for Continuous Synthesis and Processing (CCFLOW), Research Center Pharmaceutical Engineering (RCPE), Inffeldgasse 13, A-8010 Graz, Austria

*E-mail: sandor.oetvoes@uni-graz.at (SBÖ); oliver.kappe@uni-graz.at (COK)

Table of Contents

1. General information S2
2. Synthesis of catalyst **4** S3
3. Synthesis of **2**: Organocatalytic conjugate addition S4
3.1. Initial batch experiments S4
3.2. Continuous flow experiments with CH_2Cl_2 as solvent S5
3.3. Comparison of catalyst swelling in different solvents S6
3.4. Continuous flow experiments under solvent-free conditions S7
3.5. Large-scale continuous flow synthesis of **2** under solvent-free conditions S8
4. Synthesis of lactam **3**: Tandem reductive amination–lactamization S10
5. Synthesis of phenylpiperidin **1**: BH_3-mediated amide/ester reduction S13
6. Telescoped flow synthesis S15
7. Characterization data S18
8. Collection of NMR spectra and HPLC chromatograms S19
9. References S31

1. General information

All solvents and chemicals were obtained from commercial vendors (Sigma-Aldrich, TCI, Alfa Aesar, or VWR) and were used as received, without further purification. Continuous flow equipment was assembled from commercially available components as detailed in Sections 3–6.

Chromatographic purification was carried out by using a Biotage Isolera automated flash chromatography system with cartridges packed with KP-SIL, 60 Å (32–63 µm particle size). Analytical thin-layer chromatography (TLC) were performed on Merck silica gel 60 GF254 plates. Compounds were visualized by means of UV or $KMnO_4$.

^{1}H-, ^{13}C- and ^{19}F-NMR spectra were recorded on a Bruker Avance III 300 MHz instrument at ambient temperature, in $CDCl_3$ as solvent, at 300 MHz, 75 MHz and 282 MHz, respectively. Chemical shifts (δ) are reported in ppm using TMS as internal standard. Coupling constants are given in Hz units.

The *ee* of the compounds was determined by using a Shimadzu HPLC system (DGU-14A degasser, SCL-10A VP system controller, SPD-10 UV-VIS detector, LC-20AT pumps) and a Chiralpak® AD-H chiral column with isocratic mixtures of hexane and *i*PrOH as eluent. Chromatographic conditions are listed in Section 7. A racemic reference sample of **2** was prepared by using a 1:1 mixture of (*R*)- and (S)-α,α-diphenyl-2-pyrrolidinemethanol trimethylsilyl ether as catalyst as follows. A mixture containing 1 equiv dimethyl malonate ($c_{malonate}$= 0.5 M), 2 equiv 4-fluorocinnamaldehyde, 0.3 equiv AcOH and 10 mol% catalyst was stirred for 24 h at RT in MeOH as solvent. The crude product was purified chromatographically using a mixture of ethyl acetate/40-60 petroleum ether as eluent. Racemic samples of **3** and **1** were prepared by means of standard flow procedures starting from (*rac*)-**2**.

Optical rotation was measured in $CHCl_3$ (HPLC-grade) at 20 or 25 °C against the sodium D-line (λ= 589 nm) on a Perkin Elmer Polarimeter 341 using a 10-cm pathlength cell.

High resolution mass spectra of pure substances were recorded in positive mode on an Agilent 6230 TOF LC/MS (G6230B) by flow injections (1 µL) on an Agilent 1260 Infinity Series HPLC (HiP Degasser G4225A, Binary Pump G1312B, ALS Autosampler G1329B, TCC Column thermostat G1316A, DAD Detector G4212B). The solvent was 50 % H_2O (+ 0.1 % 5 M ammonium formate solution) and 50 % MeOH (+ 0.1 % of a 5 M ammonium formate solution) at a flow rate of 0.3 mL·min^{-1}. A Dual AJS ESI source was used with the following settings: Gas temperature (N_2) 350 °C, drying gas (N_2): 10 L·min^{-1}; nebulizer: 40 psig; fragmentor voltage: 200 V; skimmer voltage: 65 V, OCT 1 RF Vpp: 750 V; Vcap: 3500 V; nozzle voltage: 1100 V; reference mass: 121.050873 and 922.009798. The scan range was 100–1100 m/z and 1 spectra per second was recorded.

IR spectra were recorded on a Bruker Tensor 27 / Diamond ATR FT-IR spectrometer.

Elemental analyses of catalyst **4** were performed on a LECO CHNS 932 micro-analyzer at the Universidad Complutense de Madrid, Spain.

Pt contents were determined by means of ICP-MS using an Agilent 7700x instrument. For the analysis, the samples were digested by using an MLS ultraCLAVE system (program: ramp in 30 min to 250 °C and then heating for 30 minutes at 250°C).

LC-MS analyses were performed on a Shimadzu HPLC system (DGU20A degasser, SIL-20A autosampler, CTO20A column oven, LC-20AD pumps) using a Macherey-Nagel Nucleodur C18 HTec column (150 mm × 4.6 mm, particle size 5 µm) at 37 °C with mobile phases *A* (H_2O/acetonitrile 9:1 v/v + 0.1% TFA) and *B* (acetonitrile + 0.1% TFA) at a flow rate of 0.6 mL·min^{-1}). The detection of compounds was accomplished by a diode array detector (SPDM20A) prior electrospray ionization (ESI) using a Shimadzu LCMS-QP2020 instrument. The ESI-MS was operating either in positive or negative mode with in a scan range of 100–400 m/z or 350–750 m/z.

The E-factor was calculated by dividing the mass of waste generated by the mass of product formed. The mass of the waste did not include the water.

2. Synthesis of catalyst 4

4

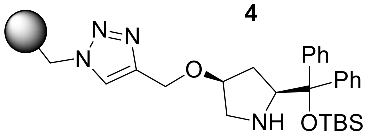

Catalyst **4** was immobilized on a cross-linked polystyrene resin (100-200 mesh) through a 1,2,3-triazole linker. The monomer synthesis and the azide–alkyne cycloaddition-based immobilization was carried out according to a recently published procedure.[1,2]

The level of functionalization of the polystyrene-supported catalyst *f* (mmol of monomeric catalyst / gram of resin) was calculated based on the results of nitrogen elemental analysis by the following formula:[3]

f (mmol g^{-1}) = %N × 1000 × (number of N atoms)$^{-1}$ × $M_W(N)^{-1}$ × 100^{-1}

Elemental analysis: N 2.60, C 86.36, H 7.59

f= 0.464 mmol g^{-1} (Complete functionalization.)

The supported material was checked by means of IR (ATR):

v= 3060, 3025, 2923, 2852, 1601, 1492, 1451, 1252, 1068, 1027, 835, 755, 695, 539 cm^{-1}

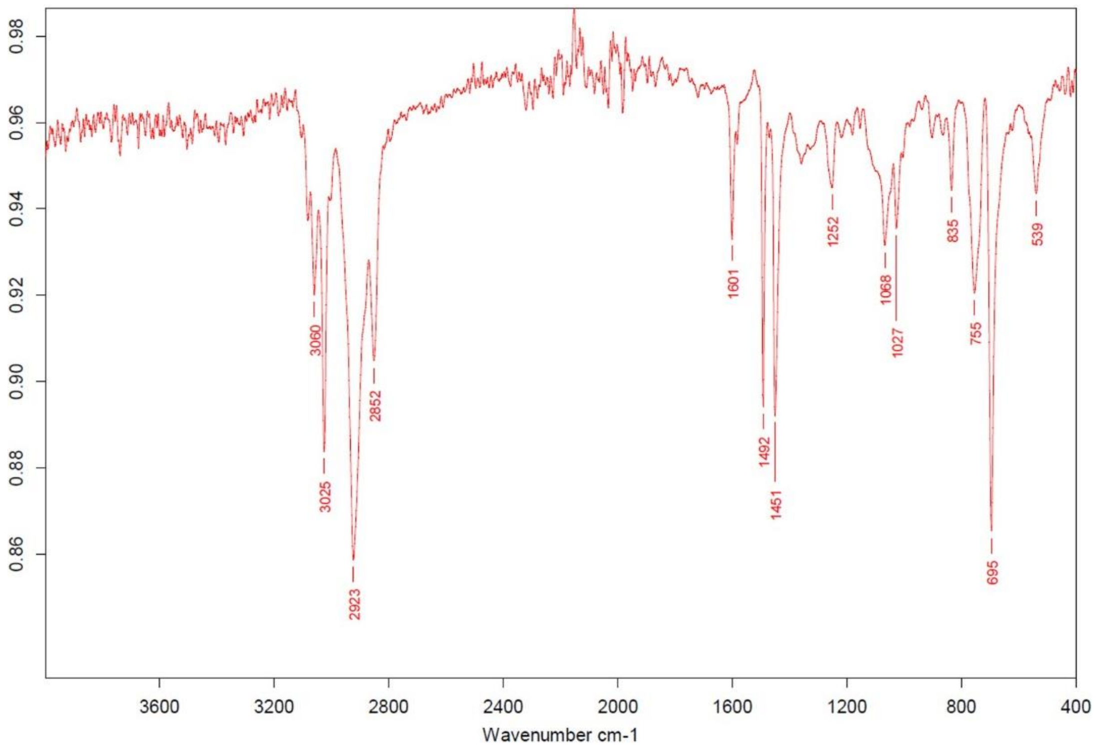

3. Synthesis of 2: Organocatalytic conjugate addition

3.1. Initial batch experiments

A typical procedure for the batch reactions is as follows: a 1-mL mixture containing 4-fluorocinnamaldehyde (1 equiv, 26.2 or 52.4 μL, 0.2 or 0.4 M), dimethyl malonate, a specified solvent and, occasionally, 0.6 equiv. of an additive was added into a glass vial. Catalyst **4** was next added (f= 0.464 mmol g^{-1}, 86 or 172 mg, 20 mol % loading), and the suspension was shaken for 24 h at 25, 50 or 75 °C. The mixture was filtered and the resin beads were washed with the same solvent used as reaction medium (5 × 1 mL). The solvent was concentrated under reduced pressure and the crude product was analyzed by means of ^{1}H-NMR and chiral HPLC.

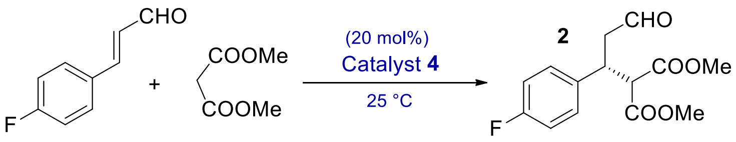

Table S1. Effects of different solvents.[a]

#[b]	Solvent	Conversion (%)[c]	*ee*[d]
1	CH_2Cl_2	45	97
2	$CHCl_3$	29	98
3	EtOH	60	95
4	MeCN	33	98
5	EtOAc	32	97
6	THF	30	96
7	2-MeTHF	37	97
8	Acetone	51	96
9	Toluene	59	95
10	DMF	77	94

[a]$c_{aldehyde}$= 0.2 M, 3 equiv. dimethyl malonate, no additives. [b]No side product formation, chemoselectivity was 100% in all reactions. [c]Determined by ^{1}H-NMR analysis of the crude product. [d]Determined by chiral HPLC.

Table S2. Effects of concentration and malonate excess.[a]

#[b]	$c_{aldehyde}$ (M)	Malonate amount (equiv.)	Conversion (%)[c]	*ee*[d]
1	0.2	2	33	97
2	0.2	3	45	97
3	0.2	9	71	98
4	0.4	9	99	97

[a]Solvent: CH_2Cl_2, no additives. [b]No side product formation, chemoselectivity was 100% in all reactions. [c]Determined by ^{1}H-NMR analysis of the crude product. [d]Determined by chiral HPLC.

Table S3. Effects of different additives.[a]

#[b]	Additive (0.6 equiv)	Conversion (%)[c]	*ee*[d]
1	-	45	97
2	LiOAc	78	97
3	AcOH	69	98
4	benzoic acid	49	97
5	picric acid	traces	not determined
6	TFA	traces	not determined

[a] $c_{aldehyde}$= 0.2 M, 3 equiv. dimethyl malonate, solvent: CH_2Cl_2. [b] Entries 1–4: no side product formation, chemoselectivity was 100%; entries 5 and 6: chemoselectivity was not determined. [c] Determined by ^{1}H-NMR analysis of the crude product. [d] Determined by chiral HPLC.

3.2. Continuous flow experiments using CH_2Cl_2 as solvent

A typical procedure for the continuous flow experiments is as follows: the reaction mixture consisting of 4-fluorocinnamaldehyde (1 equiv.), dimethyl malonate and AcOH in CH_2Cl_2 was pumped by using a Syrris® Asia syringe pump. 1 g of catalyst **4** (*f*= 0.464 mmol g^{-1}) was encompassed in an adjustable Omnifit® glass column (10 mm ID), which was heated by a Syrris® column heater. The system was pressurized by applying a 10-bar BPR from IDEX. Prior to the reactions, the catalyst was swollen by pumping CH_2Cl_2 at 200 µL min^{-1} for 45 min. (The swollen bed was approximately 7 cm high). For the reactions, the flow rate was set to 100 µL min^{-1}, which corresponded to 35 min residence time on the catalyst bed. In each runs, 2 mL product solution was collected after reaching steady state, which was next concentrated under reduced pressure and analyzed by ^{1}H-NMR and chiral HPLC.

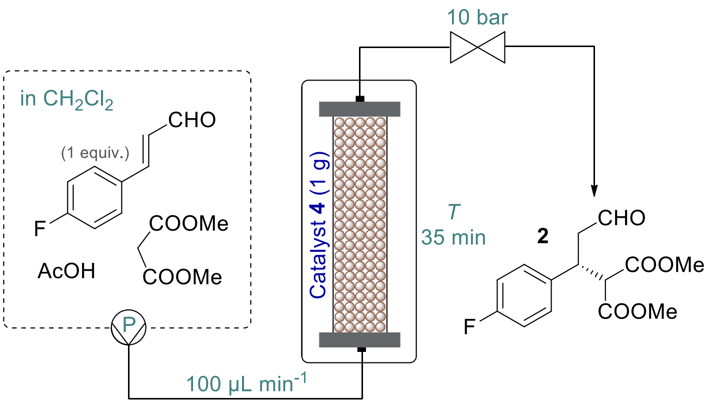

Table S4. Effects of AcOH amount.[a]

#[b]	AcOH amount (equiv.)	Conversion (%)[c]	*ee*[d]
1	0	8	98
2	0.3	14	98
3	0.6	20	98
4	1.2	18	97
5	2.0	19	97

[a] $c_{aldehyde}$= 0.4 M, 3 equiv. dimethyl malonate, at 25 °C. [b] No side product formation, chemoselectivity was 100% in all reactions. [c] Determined by ^{1}H-NMR analysis of the crude product. [d] Determined by chiral HPLC.

Table S5. Effects of reaction temperature.[a]

#[b]	T (°C)	Conversion (%)[c]	*ee*[d]
1	25	20	98
2	40	57	98
3	50	68	97

[a]$c_{aldehyde}$= 0.4 M, 3 equiv. dimethyl malonate, 0.6 equiv. AcOH as additive. [b]No side product formation, chemoselectivity was 100% in all reactions. [c]Determined by ^{1}H-NMR analysis of the crude product. [d]Determined by chiral HPLC.

Table S6. Effects of concentration and malonate excess.[a]

#[b]	$c_{aldehyde}$ (M)	Malonate amount (equiv.)	Conversion (%)[c]	*ee*[d]
1	0.1	3	6	not determined
2	0.4	3	20	98
3	0.4	9	51	98
4	0.4	15	64	99
5	0.8	3	32	98
6	1.2	3	34	98
7	1.6	3	39	98

[a]0.6 equiv. AcOH as additive, at 25 °C. [b]No side product formation, chemoselectivity was 100% in all reactions. [c]Determined by ^{1}H-NMR analysis of the crude product. [d]Determined by chiral HPLC.

3.3. Comparison of catalyst swelling in different medium

Swelling properties of resin-supported catalyst **4** were compared in CH_2Cl_2 and in dimethyl malonate. To this end, 1 g of the material was loaded into an adjustable Omnifit® glass column (10 mm ID) and was swollen by pumping the appropriate liquid at 200 µL min^{-1} for 45 min. In Figure S1, the difference in bed height represents the different swelling of the resin, which had significant effects on the residence times measured.

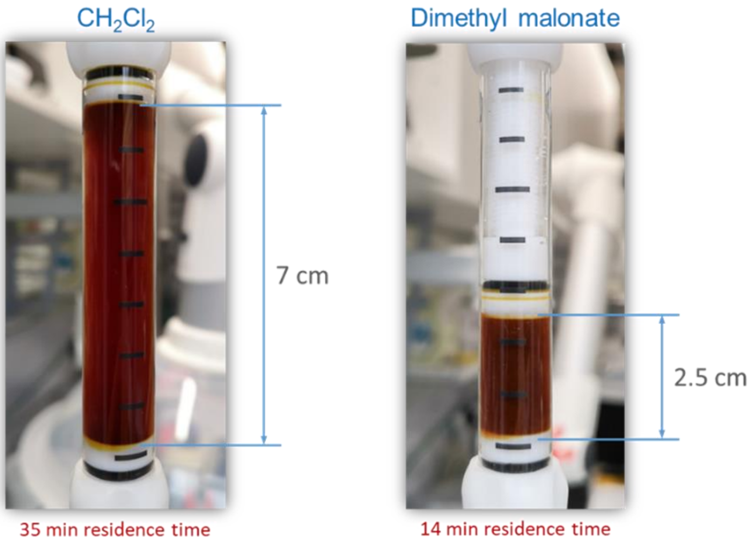

Figure S1. Swelling test of catalyst **4** in in CH_2Cl_2 and in dimethyl malonate. (Residence times were measured at 100 µL min^{-1} flow rate.)

3.4. Continuous flow experiments under solvent-free conditions

A typical procedure for the continuous flow experiments is as follows: the reaction mixture consisting of 4-fluorocinnamaldehyde (1 equiv.), dimethyl malonate and AcOH was pumped by using a Syrris® Asia syringe pump. 1 g of catalyst **4** (f= 0.464 mmol g^{-1}) was filled into an adjustable Omnifit® glass column (6.6 mm ID) which was heated by a Syrris® column heater. The system was pressurized by applying a fixed-pressure BPR from IDEX. Prior to the reactions, the catalyst was swollen by pumping dimethyl malonate at 200 µL min^{-1} for 45 min. (The swollen bed was approximately 7 cm high). In each run, 2 mL product solution was collected after reaching steady state, which was next concentrated under reduced pressure and analyzed by ^{1}H-NMR and chiral HPLC.

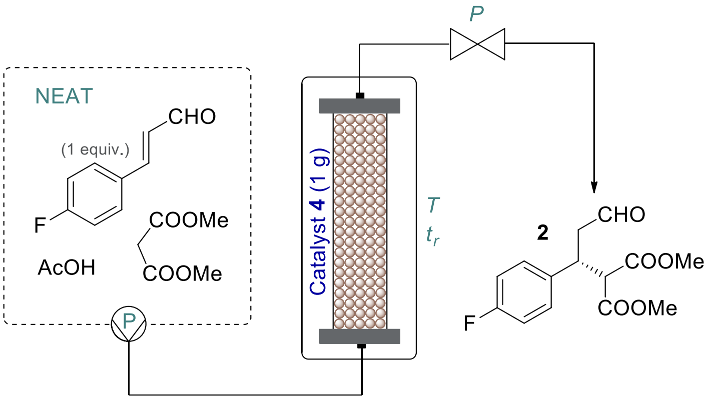

Table S7. Effects of AcOH amount and malonate excess.[a]

#[b]	Malonate amount (equiv.)	AcOH amount (equiv.)	Conversion (%)[c]	*ee*[d]
1	2	0.6	74	98
2	3	0.3	67	98
3	3	0.6	81	98
4	3	1.2	79	99
5	9	0.6	91	98

[a]Solvent-free, 100 µL min^{-1} flow rate (t_r= 14 min), T= 50 °C, P= 10 bar. [b]No side product formation, chemoselectivity was 100% in all reactions. [c]Determined by ^{1}H-NMR analysis of the crude product. [d]Determined by chiral HPLC

Table S8. Effects of pressure and reaction temperature.[a]

#[b]	*T* (°C)	*P* (bar)	Conversion (%)[c]	*ee*[d]
1	50	3	78	98
2	50	10	81	98
3	50	17	82	98
4	60	10	89	97
5	70	10	94	95
6	80	10	100	94

[a]3 equiv. dimethyl malonate, 0.6 equiv. AcOH as additive, solvent-free, 100 µL min^{-1} flow rate (t_r= 14 min). [b]No side product formation, chemoselectivity was 100% in all reactions. [c]Determined by ^{1}H-NMR analysis of the crude product. [d]Determined by chiral HPLC.

Table S9. Final tweaks – effects of residence time.[a]

#[b]	Malonate amount (equiv.)	Flow rate (μL min^{-1})	t_r (min)	Conversion (%)[c]	*ee*[d]
1	3	100	14	89	97
2	3	70	20	95	97
3	3	50	28	96	97
4	2	100	14	84	97
5	2	70	20	93	97
6	2	50	28	94	97
7	2	20	70	99	95

[a]0.6 equiv. AcOH as additive, solvent-free, *T*= 60 °C, *P*= 10 bar. [b]No side product formation, chemoselectivity was 100% in all reactions. [c]Determined by ^{1}H-NMR analysis of the crude product. [d]Determined by chiral HPLC.

Taking into account the *ee*, conversion and maximum attainable productivity, the conditions in Table S9, entry 5 were designated as optimum and applied in the subsequent preparative scale synthesis.

*3.5. Large-scale continuous flow synthesis of **2** under solvent-free conditions*

The procedure for the large-scale asymmetric flow synthesis of **2** is as follows. The reaction mixture consisting of 4-fluorocinnamaldehyde (1 equiv.), dimethyl malonate (2 equiv.) and AcOH (0.6 equiv.) was pumped by using a Syrris® Asia syringe pump. (In this reaction mixture, the concentration of 4-fluorocinnamaldehyde was 2.48 M as determined experimentally.) 1 g of catalyst **4** (*f*= 0.464 mmol g^{-1}) was filled into an adjustable Omnifit® glass column (6.6 mm ID) which was heated by a Syrris® column heater at 60 °C. The system was pressurized by applying a 10-bar BPR from IDEX. Prior to the reaction, the catalyst was swollen by pumping dimethyl malonate at 200 μL min^{-1} for 45 min. (The swollen bed was approximately 7 cm high). The flow rate was set to 70 μL min^{-1} (corresponded to 20 min residence time on the catalyst bed), and the product stream was collected continuously for 7 h after reaching steady state. During this period, samples were taken in every 15 minutes and conversion, chemoselectivity and *ee* were determined in all of them by means of ^{1}H-NMR and chiral HPLC, respectively.

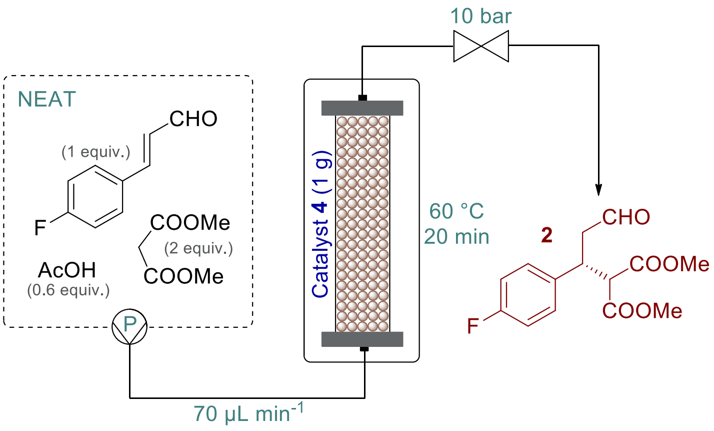

According to the analysis of the samples collected, catalyst **4** proved highly robust during the large scale solvent-free run. The selectivity of the catalyst remained unchanged: *ee* was constant in the range of 95–98%; side products were not detected, i.e. chemoselectivity was 100%. Only a small decrease in catalytic activity occurred during the experiment as indicated by a slight drop in conversion from 93 to 85%. Conversion and *ee* are represented as functions of time-on-stream in Figure S2.

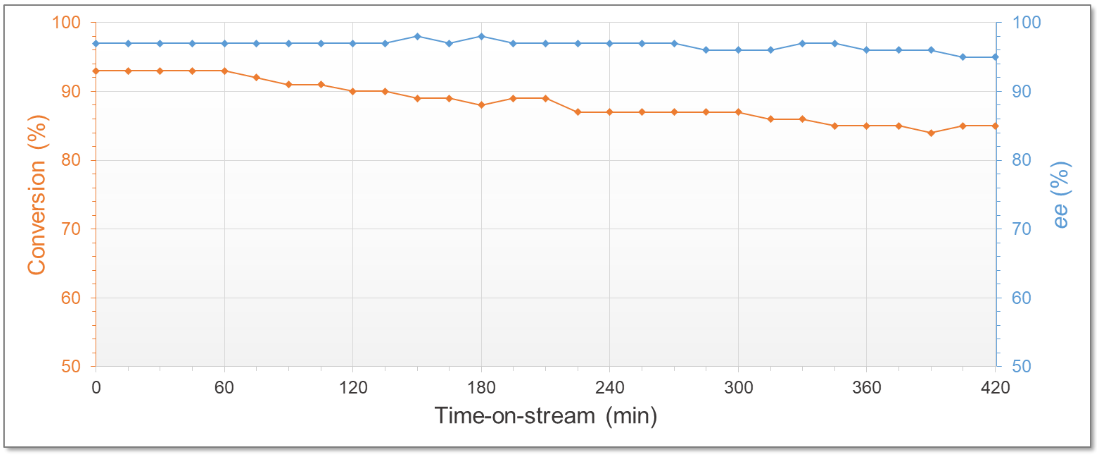

Figure S2. Investigation of stability of catalyst **4** during the large-scale continuous flow synthesis of **2** under solvent-free conditions.

After the collection period, excess dimethyl malonate, unreacted aldehyde and residual AcOH could simply be removed *in vacuo* (55 °C, 10^{-3} mbar) yielding 17.26 g analytically pure **2** without the need for chromatographic purification (84% isolated yield). The results of the experiment are summarized below in Table S10. Picture of the flow setup can be found as Figure S3.

Table S10. Summary of the 7 h long large-scale continuous flow synthesis of **2** (Figure S2).

Amount of isolated product	17.26 g
Isolated yield	84%
Productivity	2.47 g h^{-1} of pure product
ee	97%
Effective catalyst loading for the experiment	0.6 mol%
Turnover number for the experiment	132

The same batch of catalyst was reused in two more preparative-scale runs to accumulate **2** for the optimization of the next step. Conversion and selectivity were not monitored regularly, but yield and *ee* were determined as follows: 75% yield and 96% *ee* in the first prep. run (7 h long), 64% yield and 95% *ee* in the second prep. run (5 h long).

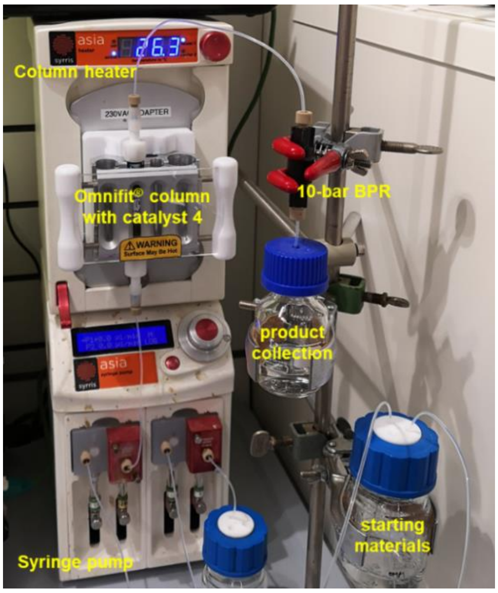

Figure S3. Picture of the continuous flow setup.

4. Synthesis of lactam 3: tandem reductive amination–lactamization

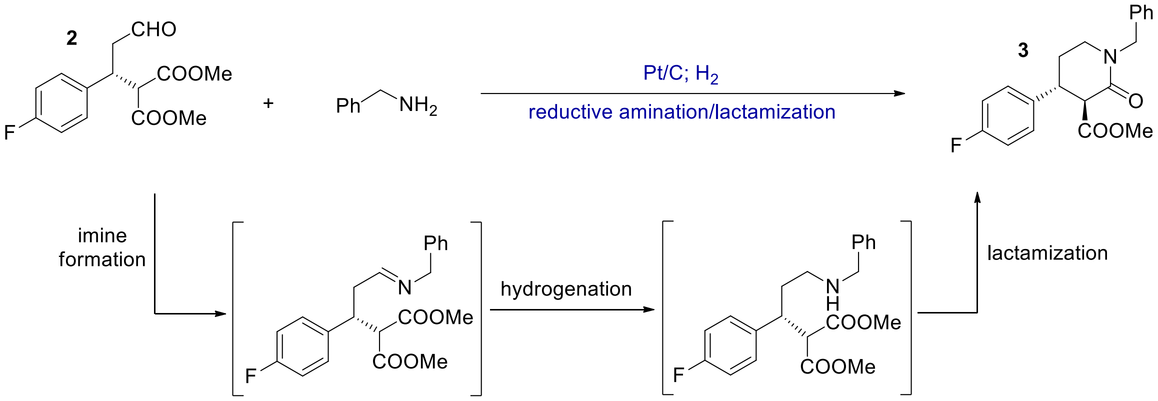

Solutions of **2** and benzylamine were pumped as separate feeds (P1 and P2) by using a UNIQSIS Binary Pump Module and were combined in a Y-mixer. The module was equipped with two high-pressure HPLC pumps, two injection valves with sample loops and a pressure sensor to monitor system pressure. H_2 gas was introduced into the system from a gas cylinder using a calibrated mass flow controller (MFC, Bronkhorst-EL). The inclusion of a check valve prevented any backflow of liquid towards the MFC. The gas flow rate was measured in units of mL_n min^{-1} (n represents measurement under standard conditions: T_n= 0 °C, P_n = 1.01 bar). The liquid and gaseous streams were combined in a second Y-mixer at room temperature. A stainless steel column with internal dimensions of 4.6 × 100 mm was used as catalyst bed and was charged with a mixture of 200 mg of 5% Pt/C and 400 mg of activated charcoal. The packed column was sealed with compatible frits (0.5 µm pore size), and was placed into a Phoenix Flow Reactor™ (ThalesNano) for heating purposes. Prior to the catalytic reactions, dry THF was pumped through the packed bed at 200 µL min^{-1} for 45 min to remove water traces from the catalyst. The flow system was pressurized by applying a fixed-pressure BPR from IDEX.

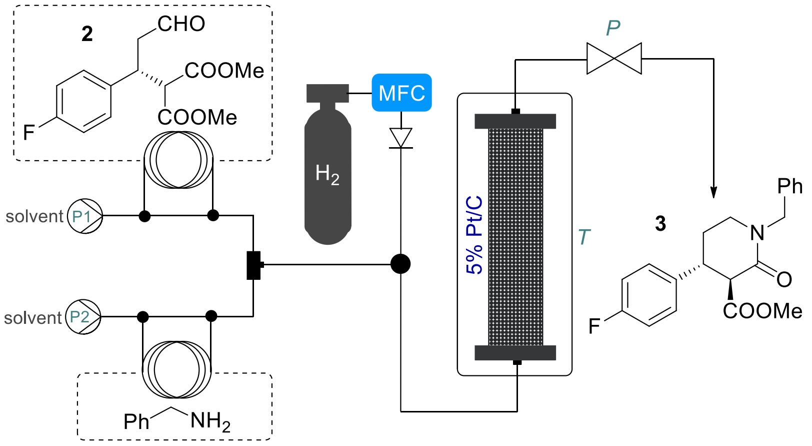

A typical procedure for the continuous flow experiments is as follows. First, the carrier solvent flow was started. Then, when the pressure stabilized on the catalyst bed, the desired temperature was set on the Phoenix Flow Reactor™ and the gas flow was initiated by setting the desired flow rate on the MFC. Once a stable segmented flow regime was observed and the pressure and the temperature of the reactor were stabilized, the system was ready for feed injection. For small-scale reactions (parameter optimization), the starting material solutions (prepared under Ar atmosphere) were injected by using 2-mL sample loops (PFA tubing, 1/16” OD, 0.80 mm ID). In each runs, the product stream was collected for 5 min after reaching steady state, which was next concentrated under reduced pressure and analyzed by ^{1}H-NMR and chiral HPLC.

<u>CAUTION:</u> H_2 is extremely flammable, therefore extreme care must be taken when handling. All equipment must be set up in a well-ventilated fume hood. A thorough safety assessment should be made before conducting any experiments.

Table S11. Effects of pressure and reaction temperature.[a]

#	*P* (bar)	*T* (°C)	Conversion (%)[b]	Chemoselectivity (%)[b]		*trans*/*cis*[b]	*ee*[c]
				**3**	**3a**		
1	3	25	95	42	58	56:44	95
2	3	50	98	94	6	78:22	95
3	3	80	100	100	0	89:11	96
4	3	100	100	100	0	93:7	96
5	3	120	100	100	0	93:7	96
6	3	150	100	100	0	92:8	95
7	10	25	94	53	47	64:36	96
8	10	50	100	97	3	82:18	94
9	10	100	100	100	0	93:7	95
10	10	120	100	100	0	93:7	95

[a]Solvent: toluene, c_2= 0.2 M, $c_{benzylamine}$= 0.2 M, P1 and P2 at 100 µL min^{-1}, 15 mL_n min^{-1} H_2 flow rate. [b]Determined by ^{1}H-NMR analysis of the crude product. [c]Determined by chiral HPLC.

Table S12. Effects of different solvents.[a]

#[b]	Solvent	Conversion (%)[c]	*trans*/*cis*[c]	*ee*[d]
1	toluene	100	93:7	96
2	EtOAc	100	92:8	96
3	EtOH	100	91:9	95
4	2-MeTHF	100	93:7	96

[a]c_2= 0.2 M, $c_{benzylamine}$= 0.2 M, P1 and P2 at 100 µL min^{-1}, 15 mL_n min^{-1} H_2 flow rate, *T*= 100 °C, *P*= 3 bar. [b]No side product formation, chemoselectivity was 100% in all reactions. [c]Determined by ^{1}H-NMR analysis of the crude product. [d]Determined by chiral HPLC.

Table S13. Effects of concentration and starting material ratio.[a]

#	*c* (M)		Conversion (%)[b]	Chemoselectivity (%)[b]		*trans*/*cis*[b]	*ee*[c]
	**2**	benzylamine		**3**	**3a**		
1[d]	0.2	0.2	100	100	0	93:7	96
2[d]	0.26	0.2	100	70	30	91:9	95
3[d]	0.2	0.26	100	100	0	92:8	95
4[d]	0.2	0.4	100	100	0	92:8	95
5[d]	0.2	0.6	100	100	0	93:7	94
6[d]	0.4	0.4	100	100	0	92:8	95
7[e]	1.0	1.0	100	100	0	93:7	96
8[e]	2.0	2.0	100	100	0	93:7	96

[a]P1 and P2 at 100 µL min^{-1}, 15 mL_n min^{-1} H_2 flow rate, *T*= 100 °C, *P*= 3 bar. [b]Determined by ^{1}H-NMR analysis of the crude product. [c]Determined by chiral HPLC. [d]Solvent: toluene. [e]Solvent: 2-MeTHF.

No dependence of conversion, chemo-, diastereo- or enantioselectivity was found utilizing different fluid flow rates in the range of 2 × 50–200 µL min^{-1} (P1 and P2) and different gas flow rates in the range of 5–25 mL$_n$ min^{-1} (c_2= 0.2 M, $c_{benzylamine}$= 0.2 M, T= 100 °C, P= 3 bar). These results are therefore not represented in details.

In case of lower temperatures or an excess of **2** (Table S11, entries 1, 2, 7, 8 and Table S13, entry 2), side product **3a** was formed *via* unwanted double alkyation as corroborated by ^{1}H-NMR and mass spectrometry (Figure S4).

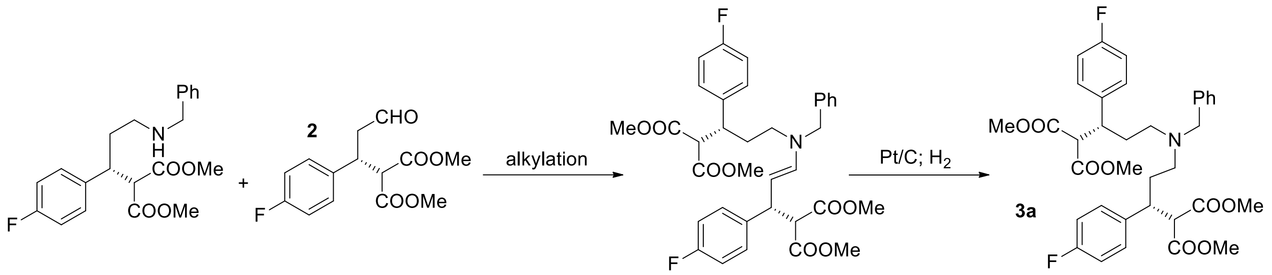

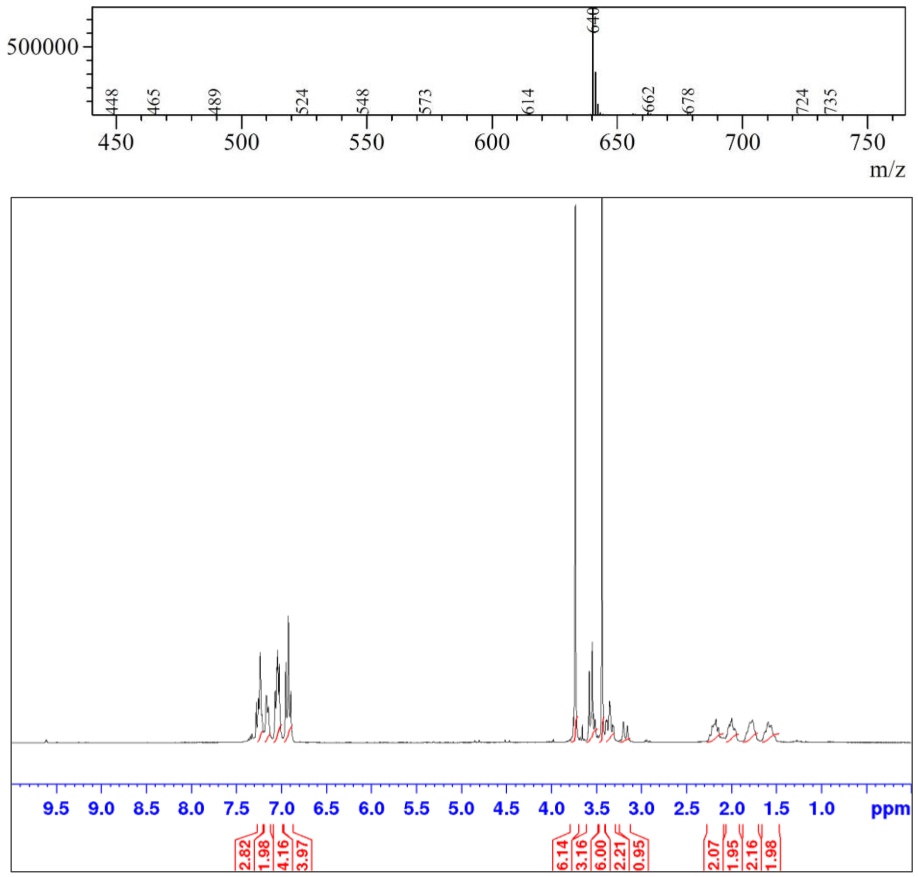

Figure S4. ^{1}H-NMR and mass spectra of side product **3a**.

In order to achieve lactam **3** in multigram scales, numerous preparative runs were carried out under optimum flow conditions (see: Table S13, entry 8). The system proved stable during the long runs, and resulted around 4 g h^{-1} of pure product with isolated yields in the range of 96–99% and *ee*s of 96%. The product obtained was sufficiently pure without chromatographic purification, and was used in the next step directly after evaporation.

According to ICP MS measurements, practically no leaching occurred from the catalyst bed during the reactions (Pt contents detected in the crude product samples were in the range of 5–6 ppb). The complete optimization study and all the preparative runs were fulfilled by a single catalyst cartridge containing merely 10 mg of Pt (200 mg 5% Pt/C). In these experiments, approximately 75 mmol substrate was transformed resulting around 25 g lactam **3**. These gave an effective catalyst loading of 0.07% and a turnover number of 1430.

5. Synthesis of phenylpiperidin 1: BH_3-mediated amide/ester reduction

The starting material solutions were pumped as separate feeds (P1 and P2) by using a UNIQSIS Binary Pump Module equipped with two high-pressure HPLC pumps, two injection valves with sample loops and a pressure sensor to monitor system pressure. The liquid streams were combined in a Y-mixer at room temperature and the resulting solution was directed through a 12-mL reaction coil (PFA tubing, 1/8" OD, 1.58 mm ID) which was heated in an oil bath. The flow system was pressurized by applying a 10-bar BPR from IDEX.

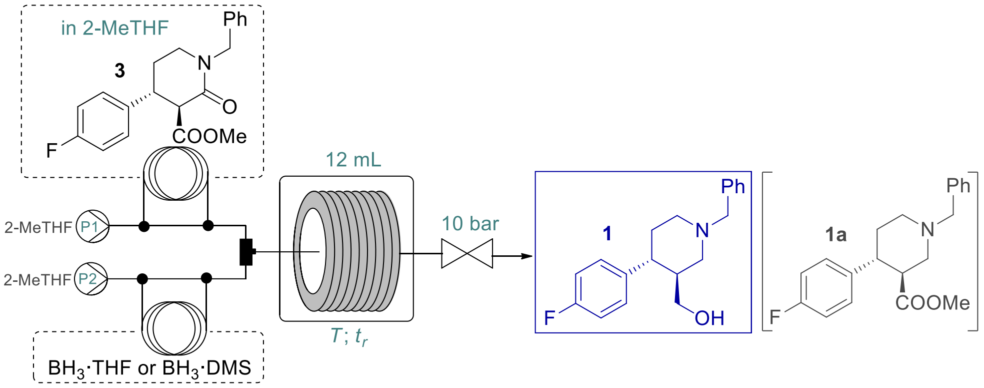

A typical procedure for the continuous flow experiments is as follows. For small-scale reactions (parameter optimization), the solution of lactam **3** in dry 2-MeTHF (prepared under Ar atmosphere) and the reducing agent were injected by using 2- or 3-mL sample loops (PFA tubing, 1/16" OD, 0.8 mm ID). Dry 2-MeTHF was used as carrier solvent. As reducing agent, commercially available BH_3·THF (1 M in THF) and BH_3·DMS (1 M in 2-MeTHF, 2 M in THF) solutions or neat BH_3·DMS (10 M) were employed. 2.5 M and 5 M BH_3·DMS solutions were prepared by dilution from the commercial reagent under Ar atmosphere. In each runs, the product stream was collected for 3–5 min after reaching steady state. In order to safely decompose the unreacted reducing agent, the stream exiting the reactor was collected into a flask containing a well-stirred 1:1 mixture of 3 M HCl and 2-MeTHF. After the collection period, the solution was refluxed for 30 min in order to remove BH_3 adducts and was next treated with NaOH solution (2 M) until pH 10. The resultant mixture was extracted three times with EtOAc. The combined organic layers were washed with brine and dried over Na_2SO_4. The filtrate was concentrated under reduced pressure and analyzed by ^{1}H-NMR and chiral HPLC.

<u>CAUTION:</u> Borane reagents are extremely dangerous. They decompose thermally or in the presence of atmospheric moisture, water and acids resulting flammable gases (B_2H_6 and H_2) and boric acid (possible blockage in reactor channels). Extreme care must therefore be taken when handling. Dry conditions must be ensured during experimentation and all equipment must be set up in a well-ventilated fume hood. A thorough safety assessment should be made before conducting any experiments.

Table S14. Preliminary experiments with BH_3·THF solution.

#[a]	*c* (M)		Flow rate (μL min^{-1})		**3** / BH_3·THF ratio	t_r (min)	*T* (°C)	Conversion (%)[c]	Chemoselectivity (%)[c]	
	**3**	BH_3·THF[b]	P1	P2					**1**	**1a**
1	0.2	1	200	200	1:5	30	25	62	66	34
2	0.2	1	200	200	1:5	30	50	99	70	30
3	0.1	1	200	200	1:10	30	50	100	73	27
4	0.1	1	100	100	1:10	60	50	100	76	24
5	0.1	1	100	100	1:10	60	70	100	94	6
6	0.1	1	100	100	1:10	60	90	100	100	0

[a]Enantiomeric purity was not affected by the reactions, 95–96% *ee* was measured in all samples (by using chiral HPLC). [b]BH_3·THF solution in THF. [c]Determined by ^{1}H-NMR analysis of the crude product.

At temperatures around 100–120 °C, occasional gas formation and precipitation occurred in the reaction coil. To ensure stable and safe operation, reaction temperature was maximized in 90 °C during parameter optimization.

Table S15. Preliminary experiments with $BH_3 \cdot DMS$ solutions.

#[a]	*c* (M)		Flow rate (µL min^{-1})		**3** / $BH_3 \cdot DMS$ ratio	t_r (min)	*T* (°C)	Conversion (%)[b]	Chemoselectivity (%)[b]	
	**3**	$BH_3 \cdot DMS$	P1	P2					**1**	**1a**
1	1.0	2.5[c]	200	200	1:2.5	30	50	77	53	47
2	0.1	1[c]	100	100	1:10	60	50	96	71	29
3	0.1	1[c]	100	100	1:10	60	70	100	92	8
4	0.1	1[c]	100	100	1:10	60	90	100	100	0
5	0.2	2[d]	100	100	1:10	60	90	100	100	0
6	0.5	5[c]	100	100	1:10	60	90	100	100	0

[a]Enantiomeric purity was not affected by the reactions, 95–96% *ee* was measured in all samples (by using chiral HPLC). [b]Determined by ^{1}H-NMR analysis of the crude product. [c]$BH_3 \cdot DMS$ solution in 2-MeTHF. [d]$BH_3 \cdot DMS$ solution in THF.

Table S16. Experiments with neat $BH_3 \cdot DMS$.[a]

#[b]	Flow rate (µL min^{-1})		**3** / $BH_3 \cdot DMS$ ratio	t_r (min)	*T* (°C)	Conversion (%)[c]	Chemoselectivity (%)[c]	
	P1	P2					**1**	**1a**
1	200	200	1:10	30	50	96	74	26
2	100	100	1:10	60	90	100	100	0
3	130	70	1:5.4	60	90	100	100	0
4	140	60	1:4.3	60	90	100	95	5
5	150	50	1:3.3	60	90	99	76	24
6	260	140	1:5.4	30	90	100	100	0
7	390	210	1:5.4	20	90	100	93	7

[a]$c_{\mathbf{3}}$= 1.0 M in 2-MeTHF, neat $BH_3 \cdot DMS$ (10 M). [b]Enantiomeric purity was not affected by the reactions, 95–96% *ee* was measured in all samples (by using chiral HPLC). [c]Determined by ^{1}H-NMR analysis of the crude product.

Under optimum flow conditions (see: Table S16, entry 6), a preparative-scale run was carried out by using neat $BH_3 \cdot DMS$ as reducing agent. For this, the product stream was collected for 30 min after reaching steady state. After extractive work-up, 2.28 g crude product was obtained. According to ^{1}H-NMR measurements, the material was acceptably pure without chromatographic purification. However, in order to remove dimethyl sulfide traces (smelly in very low concentrations), column chromatographic purification was carried out using a mixture of ethyl acetate/40-60 petroleum ether as eluent in the presence 1% trimethylamine as additive. After purification, 1.97 g of **1** was isolated (84% yield), and *ee* was 96%. The process ensured an outstanding productivity of 3.94 g h^{-1} of pure product.

6. Telescoped flow synthesis

Aldehyde **2** was obtained in continuous flow organocatalytic conjugate addition between 4-fluorocinnamaldehyde and dimethyl malonate under solvent-free conditions as described in section 3.5 and was used directly after removal of unreacted reaction components by evaporation.

The optimum conditions (Table S13, entry 8 and Table S16, entry 6) determined during the step-by-step experiments were taken into account when designing the telescoped sequence. In order to match flow rates, the 12-mL reaction coil used for the amide/ester reduction was exchanged to a 9.3-mL one.

For the tandem reductive amination–lactamization, 2.0 M solutions of **2** and benzylamine were prepared in dry 2-MeTHF (under Ar atmosphere) and were pumped as separate feeds (P1 and P2) at 100 µL min^{-1} from 15-mL sample loops with dry 2-MeTHF as carrier solvent by using a UNIQSIS Binary Pump Module. (6-port injection valves were integrated into the pump module.) The liquid streams were combined in a Y-mixer at room temperature. H_2 gas was introduced into the system from a gas cylinder using a calibrated MFC (Bronkhorst-EL). The inclusion of a check valve prevented any backflow of liquid towards the MFC. The liquid and gaseous streams were combined in a second Y-mixer at room temperature. The resulting gas–liquid feed entered a 4.6 × 100 mm stainless steel column packed with a mixture of 200 mg of 5% Pt/C and 400 mg of activated charcoal. (Prior to the experiment, dry THF was pumped through the packed bed at 200 µL min^{-1} for 45 min to remove water traces from the catalyst.) The Pt/C column was heated at 100 °C by using a Phoenix Flow Reactor™ (ThalesNano); 3 bar was maintained by a fixed-pressure BPR from IDEX. During reductive amination, one equivalent of water is released which must be removed in order to prevent decomposition of BH_3·DMS downstream. The gas–liquid mixture exiting the Pt/C column was therefore passed through a 10 × 100 mm stainless steel column packed with 5 g of freshly activated 4 Å MS. H_2 gas was separated from the liquid mixture through a buffer flask. The dried and degassed stream containing approximately 1 M 2-MeTHF solution of lactam **3** was re-incorporated for the subsequent amide/ester reduction through a 3-port valve by using a Knauer Azura P 4.1S HPLC pump (P3) at a flow rate of 200 µL min^{-1}. Neat BH_3·DMS was streamed from a 20-mL sample loop with dry 2-MeTHF as carrier solvent by using a Knauer WellChrom K-120 HPLC pump (P4) and a manual 6-port injection valve from IDEX. Both liquid lines were pressurized by 3-bar BPRs from IDEX and were combined in a Y-mixer at room temperature. The resulting solution was directed through a 9.3-mL reaction coil (PFA tubing, 1/8” OD, 1.58 mm ID) which was heated at 90 °C in an oil bath. The system was pressurized applying a 10-bar BPR from IDEX. Pictures of the telescoped setup can be found in Figures S5 and S6.

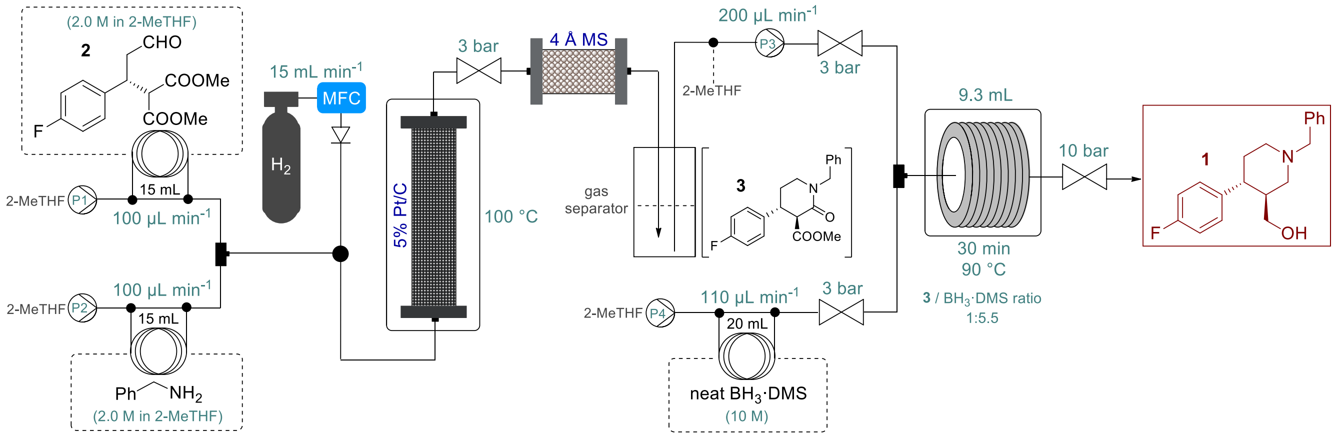

A typical procedure for the experiment is as follows: first, the carrier solvent flows were started (P1, P2, P3 and P4). When the pressure stabilized on the Pt/C column, the desired temperature was set on the Phoenix Flow Reactor™ and the gas flow was initiated by setting the desired flow rate on the MFC. At the same time, the coil reactor was also heated up. Once a stable segmented flow regime was observed after the gas–liquid mixer and the pressure and the temperature of the reactors were stabilized, the system was ready for feed injection. Solutions of **2** and benzylamine were injected first. After 15 min, the reductive amination–lactamization stream exiting the 4 Å MS column reached steady state. Then the output was placed into the gas separator, and after 5 more min, P3 was switched from 2-MeTHF to the degassed stream of **3**. Simultaneously, the neat BH_3·DMS feed was initiated by injection. The product stream exiting the heated reaction coil was collected continuously for 100 min after reaching steady state (1 h after injection of **2** and benzylamine). In order to safely decompose the unreacted

reducing agent, the outcome from the reactor was directed into a flask containing a well-stirred 1:1 mixture of 3 M HCl and 2-MeTHF. After the collection period, the solution was refluxed for 30 min in order to remove BH_3 adducts and was next treated with NaOH solution (2 M) until pH 10. The resulting mixture was extracted three times with EtOAc. The combined organic layers were washed with brine, dried over Na_2SO_4 and concentrated under reduced pressure. Column chromatographic purification was carried out using a mixture of ethyl acetate/40-60 petroleum ether as eluent in the presence 1% trimethylamine as additive. The results of the telescoped experiment are summarized below in Table S17.

Table S17. Results of the telescoped continuous flow synthesis of phenylpiperidin **1**.

Collection time	100 min
Chemoselectivity[a]	100 %
Material isolated after chromatographic purification	4.95 g
Isolated yield	83%
Productivity	2.97 g h^{-1} of pure product
ee	96%

[a]Determined by ^{1}H-NMR analysis of the crude product.

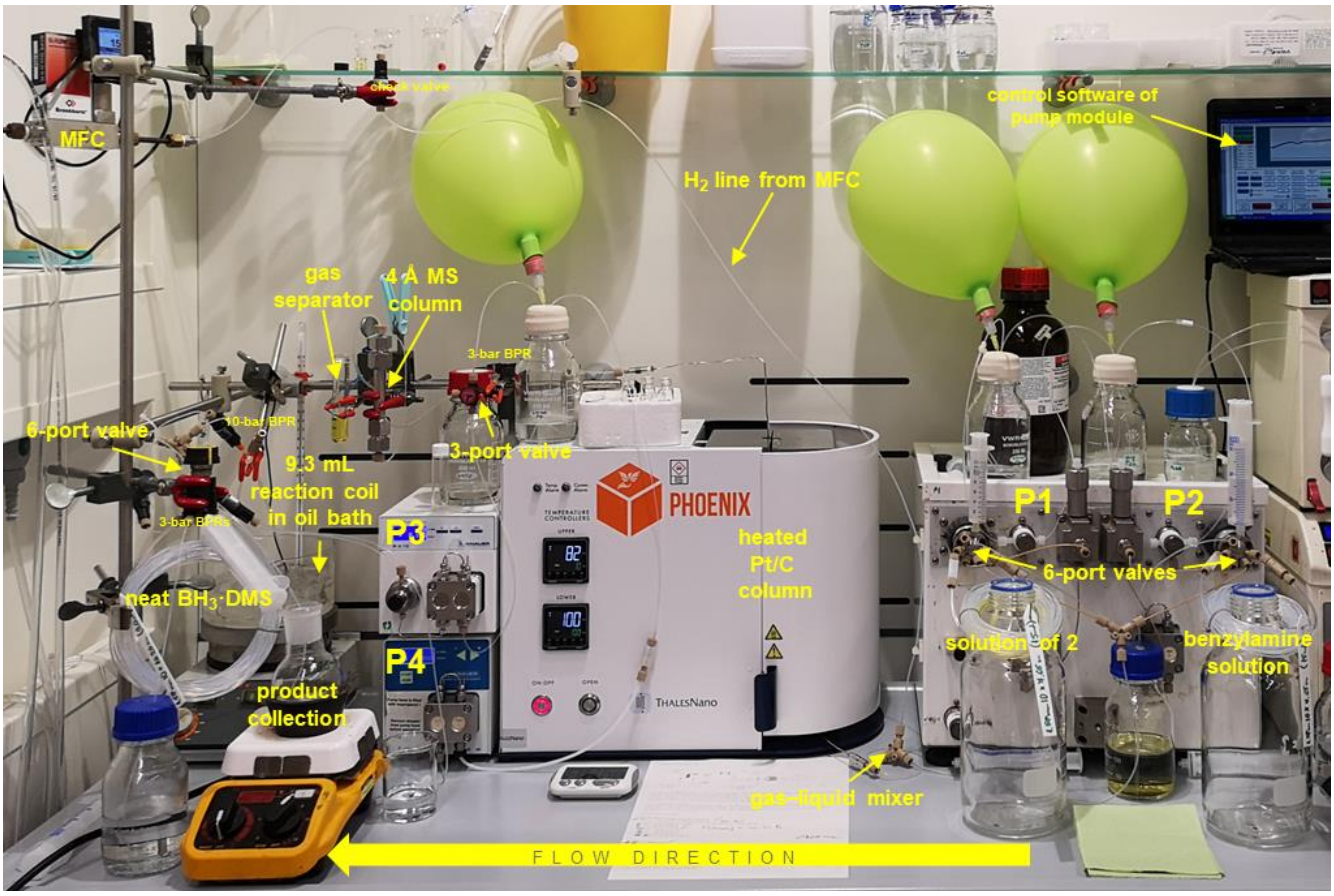

Figure S5. Continuous flow set-up for the telescoped synthesis of phenylpiperidin **1** during operation.

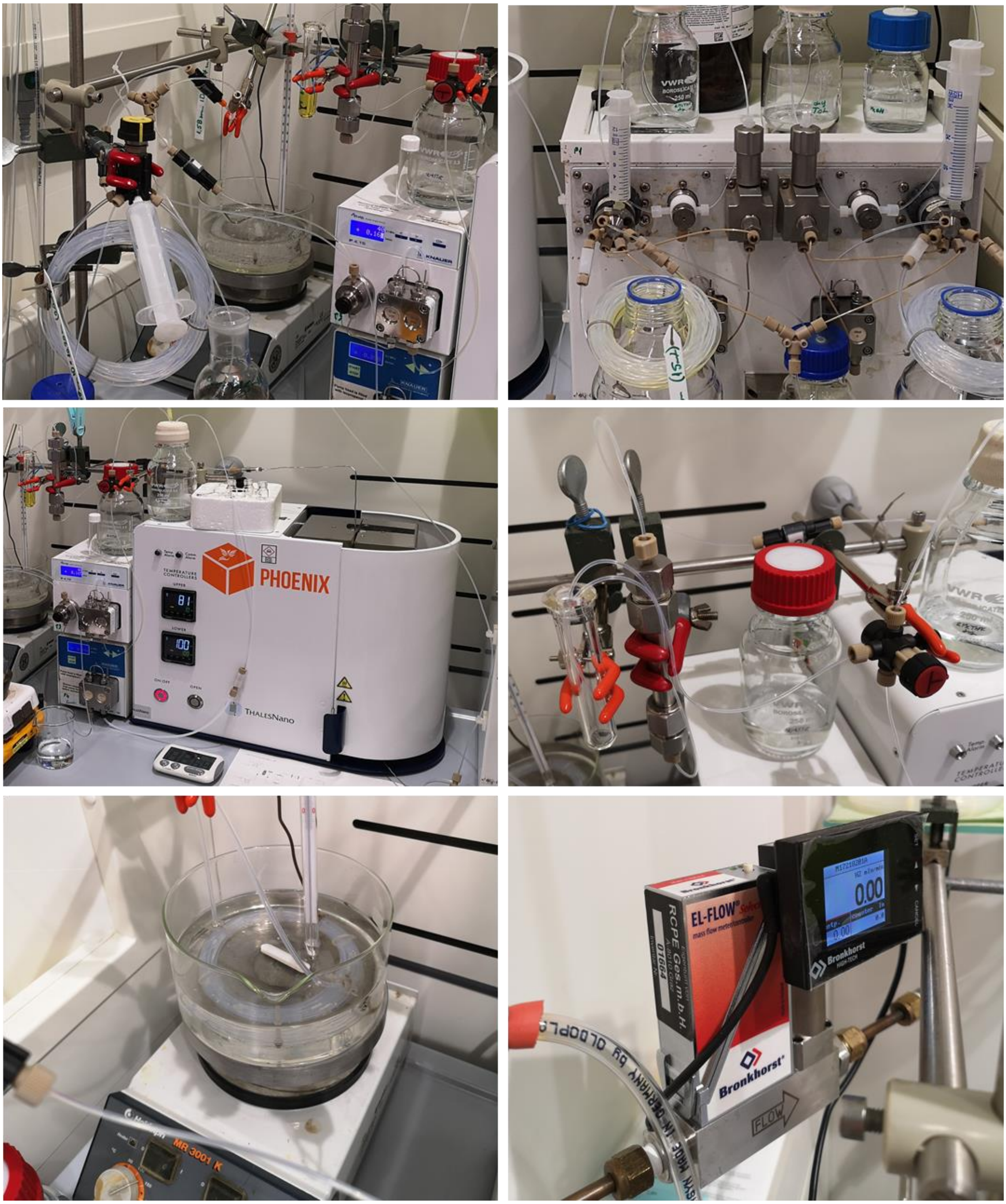

Figure S6. Details of the telescoped system.

7. Characterization data

(*R*)-2-(3-Oxo-1-(4-fluorophenyl)propyl)malonic acid dimethyl ester (**2**):

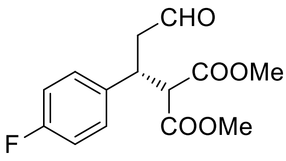

^{1}H-NMR (300 MHz, $CDCl_3$) δ 9.61 (t, *J*= 1.5 Hz, 1H), 7.27–7.20 (m, 2H), 7.00 (t, *J*= 9.0 Hz, 2H), 4.04 (dt, *J*= 8.5 Hz, *J*= 5.0 Hz, 1H), 3.80–3.68 (m, 4H), 3.53 (s, 3H), 3.03–2.82 (m, 2H); ^{13}C-NMR (75 MHz, $CDCl_3$) δ 199.6, 168.2, 167.8, 162.0 (d, *J*= 247.0 Hz), 135.5 (d, *J*= 3.3 Hz), 129.7 (d, *J*= 8.1 Hz), 115.7 (d, *J*= 21.6 Hz), 57.2, 52.8, 52.6, 47.3, 38.7; ^{19}F-NMR (282 MHz, $CDCl_3$) δ –114.7; HRMS (ESI, positive mode) calculated for $C_{14}H_{16}FO_5$ $[M+H]^+$: 283.0976, found: 283.0975; HPLC (Chiralpak® AD-H, hexane–*i*PrOH 80/20, 0.5 mL min^{-1}, 210 nm, 25 °C) t_{major}= 17.96 min, t_{minor}= 19.31 min, *ee*= 97%; $[\alpha]_D^{25}$= –29.4 (c= 1.00, $CHCl_3$) {lit.[4] $[\alpha]_D^{25}$= –30.5 (c= 1.00, $CHCl_3$)}.

(3*S*,4*R*)-1-Benzyl-4-(4-fluorophenyl)-2-oxopiperidine-3-carboxylic acid methyl ester (**3**):

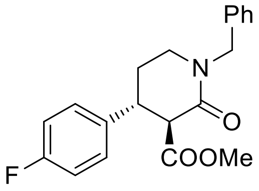

^{1}H-NMR (300 MHz, $CDCl_3$) δ 7.42–7.28 (m, 5H), 7.20–7.13 (m, 2H), 7.06–6.97 (m, 2H), 4.83 (d, *J*= 14.3 Hz, 1H), 4.49 (d, *J*= 14.3 Hz, 1H), 3.66 (s, 3H), 3.61 (d, *J*= 11.0 Hz, 1H), 3.52–3.36 (m, 2H), 3.36–3.26 (m, 1H), 2.14–1.9 (m, 2H); ^{13}C-NMR (75 MHz, $CDCl_3$) δ 170.5, 165.7, 162.0 (d, *J*= 246.0 Hz), 137.1 (d, *J*= 3.5 Hz), 136.5, 128.8, 128.3 (d, *J*= 8.6 Hz), 128.2, 127.7, 115.8 (d, *J*= 21.2 Hz), 56.6, 52.4, 50.4, 46.2, 41.7, 29.4; ^{19}F-NMR (282 MHz, $CDCl_3$) δ –115.1; HRMS (ESI, positive mode) calculated for $C_{20}H_{21}FNO_3$ $[M+H]^+$: 342.1500, found: 342.1500; HPLC (Chiralpak® AD-H, hexane–*i*PrOH 80/20, 1.0 mL min^{-1}, 210 nm, 25 °C) t_{major}= 12.52 min, t_{minor}= 16.42 min, *ee*= 96%; $[\alpha]_D^{20}$= –7.6 (c= 0.90, $CHCl_3$) {lit.[5] $[\alpha]_D^{20}$= –8.8 (c= 0.90, $CHCl_3$)}.

((3*S*,4*R*)-1-Benzyl-4-(4-fluorophenyl)piperidin-3-yl)methanol (**1**)

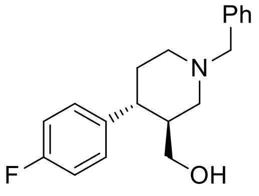

^{1}H-NMR (300 MHz, $CDCl_3$) δ 7.41–7.25 (m, 5H), 7.23–7.15 (m, 2H), 7.05–6.95 (m, 2H), 3.64 (d, *J*= 13 Hz, 1H), 3.56 (d, *J*= 13 Hz, 1H), 3.39 (dd, *J*= 11.1 Hz, *J*= 2.5 Hz, 1H), 3.29–3.16 (m, 2H), 3.04–2.93 (m, 1H), 2.42–2.29 (m, 1H), 2.12–1.95 (m, 3H), 1.92–1.72 (m, 2H), 1.41 (s, 1H); ^{13}C-NMR (75 MHz, $CDCl_3$) δ 161.8 (d, *J*= 243.0 Hz), 140.0 (d, *J*= 3.2 Hz), 137.6, 129.4, 128.8 (d, *J*= 7.8 Hz), 128.3, 127.2, 115.4 (d, *J*= 21.0 Hz), 63.8, 63.4, 57.3, 53.8, 44.1, 44.0, 34.2; ^{19}F-NMR (282 MHz, $CDCl_3$) δ –116.6; HRMS (ESI, positive mode) calculated for $C_{19}H_{23}FNO$ $[M+H]^+$: 300.1758, found: 300.1762; HPLC (derivatization with Ac_2O/DMAP, Chiralpak® AD-H, hexane–*i*PrOH 98.5/1.5, 0.5 mL min^{-1}, 210 nm, 25 °C) t_{major}= 16.22 min, t_{minor}= 18.80 min, *ee*= 96%; $[\alpha]_D^{25}$= –11.0 (c= 1.00, $CHCl_3$) {lit.[6] $[\alpha]_D^{26}$= –12.0 (c= 1.00, $CHCl_3$)}.

8. Collection of NMR spectra and HPLC chromatograms

(*R*)-2-(3-Oxo-1-(4-fluorophenyl)propyl)malonic acid dimethyl ester (**2**)

^{1}H-NMR (300 MHz, $CDCl_3$)

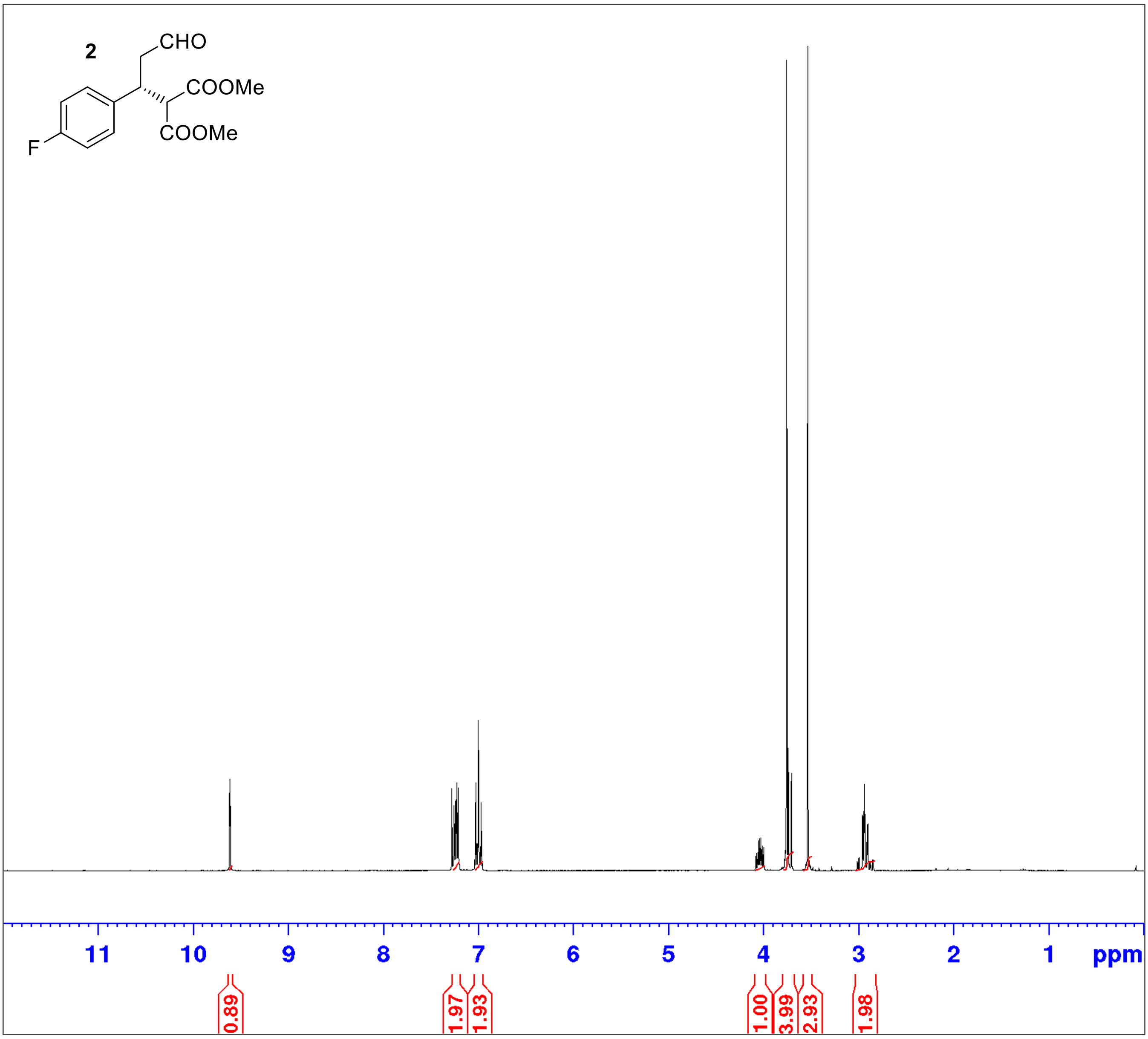

^{13}C-NMR (75 MHz, $CDCl_3$)

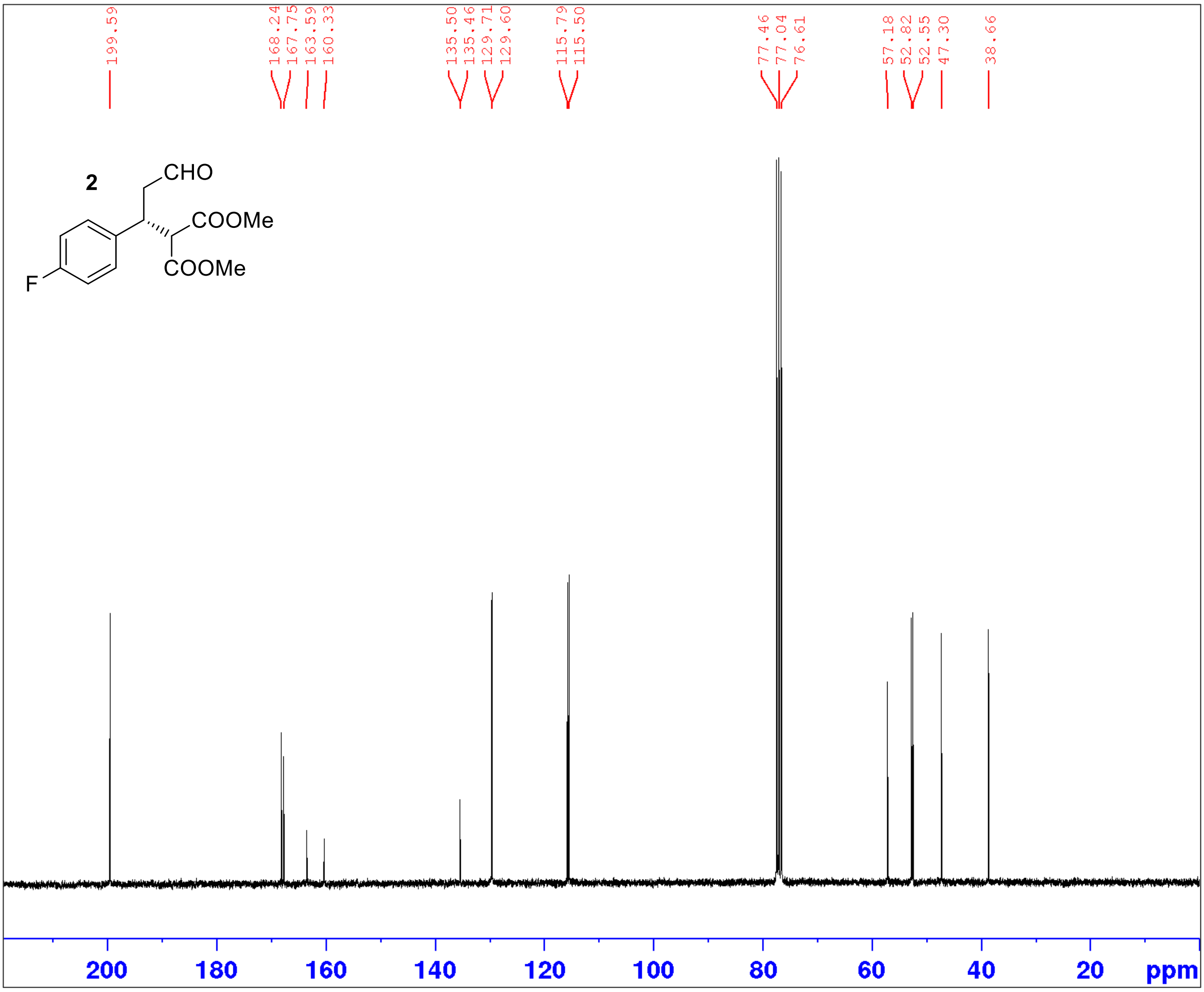

^{19}F-NMR (282 MHz, $CDCl_3$)

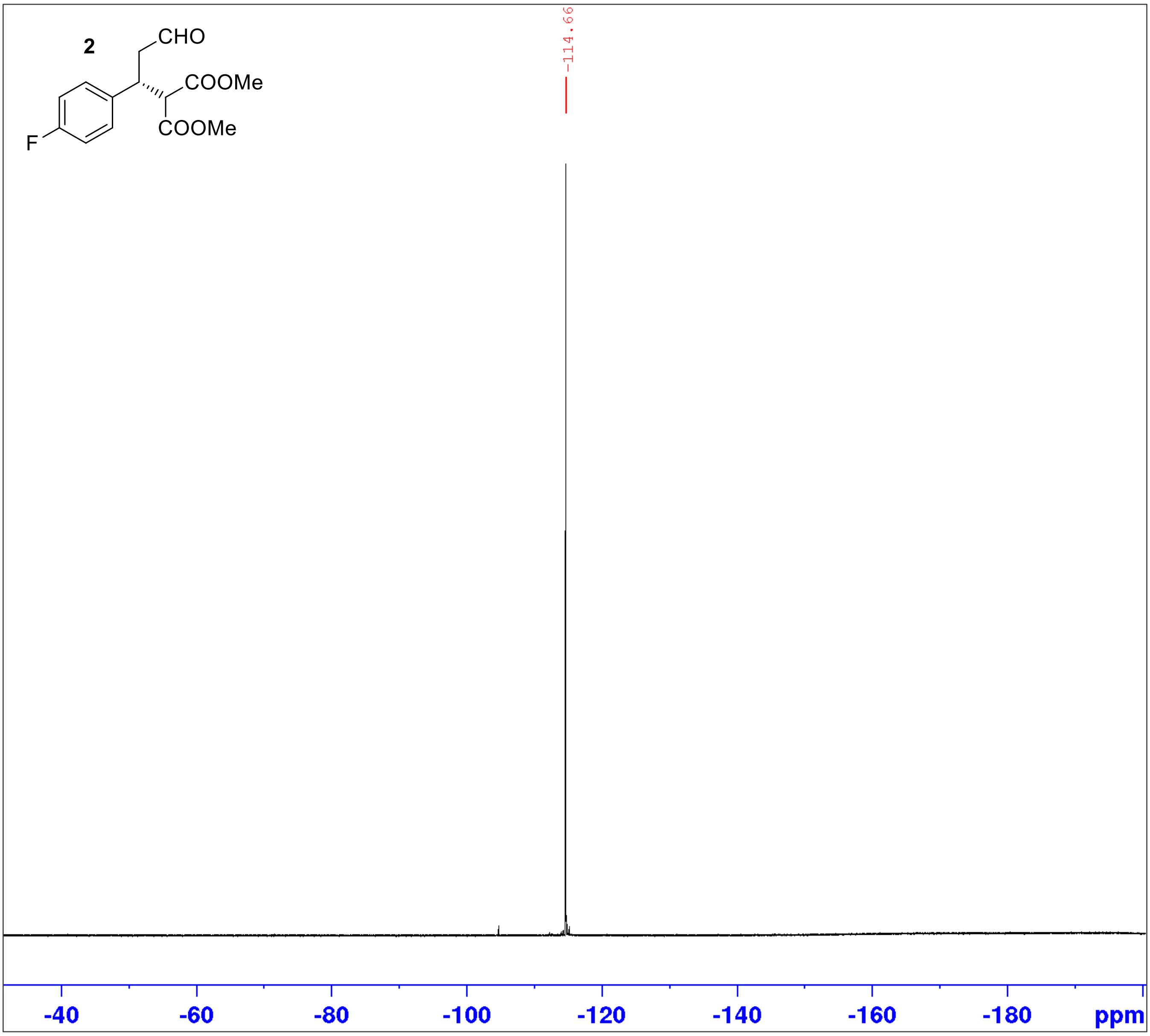

HPLC chromatograms

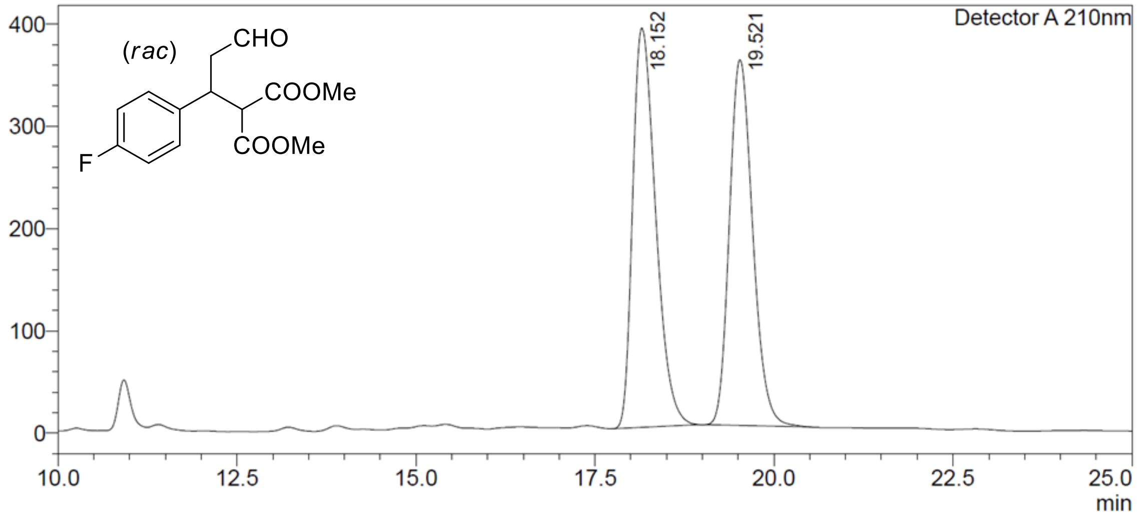

Peak#	Ret. Time	Area	Height	Conc.
1	18.152	8678703	390676	51.779
2	19.521	8082430	357903	48.221
Total		16761134	748580	

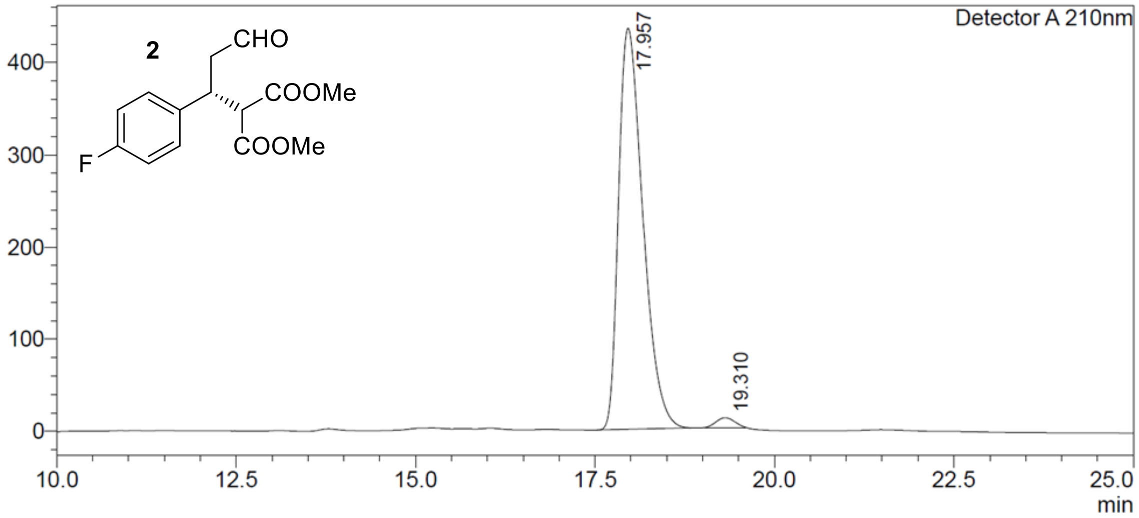

Peak#	Ret. Time	Area	Height	Conc.
1	17.957	10269576	435131	98.452
2	19.310	161455	9665	1.548
Total		10431031	444796	

(3*S*,4*R*)-1-Benzyl-4-(4-fluorophenyl)-2-oxopiperidine-3-carboxylic acid methyl ester (**3**)

^{1}H-NMR (300 MHz, CDCl$_3$)

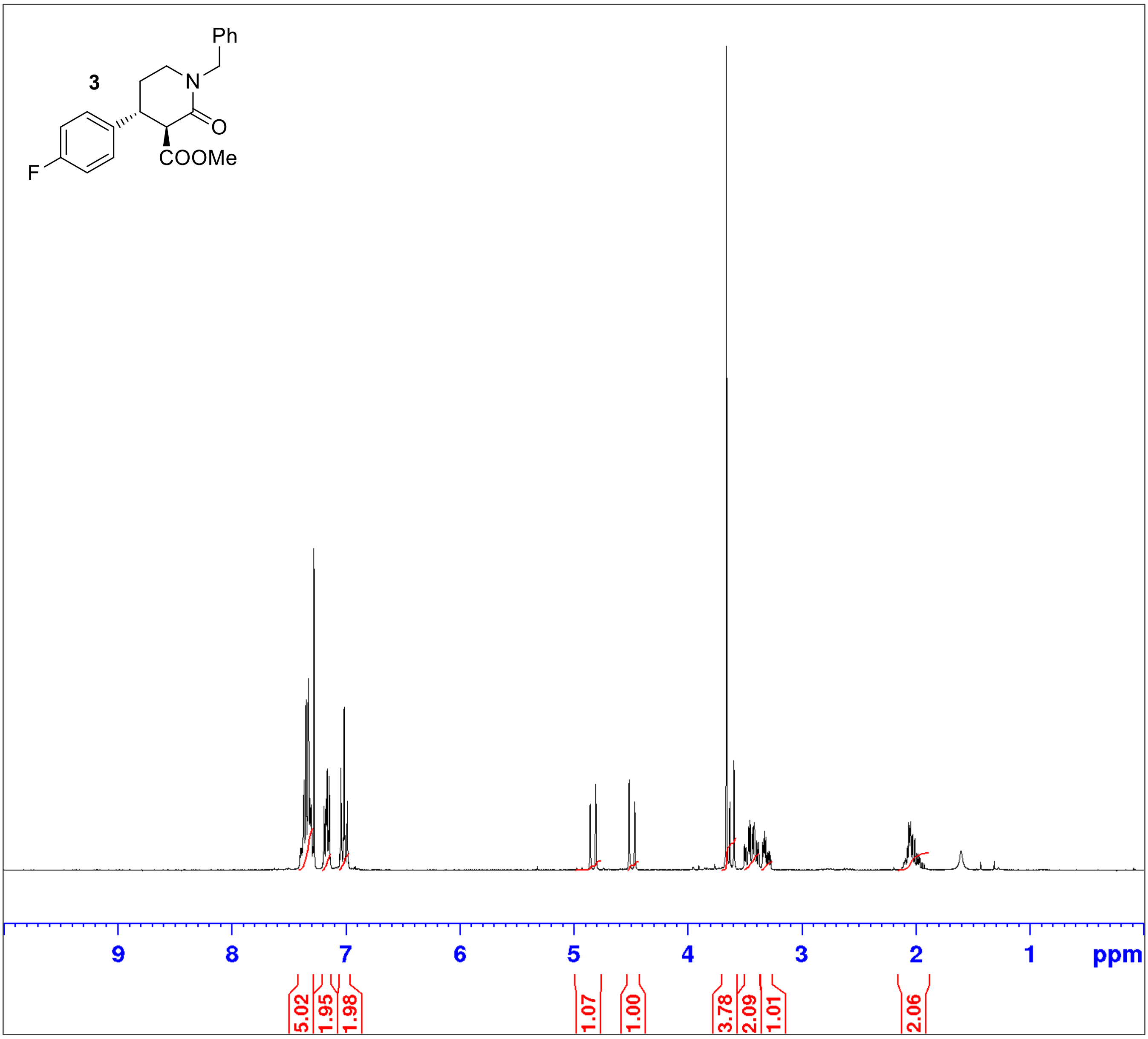

^{13}C-NMR (75 MHz, $CDCl_3$)

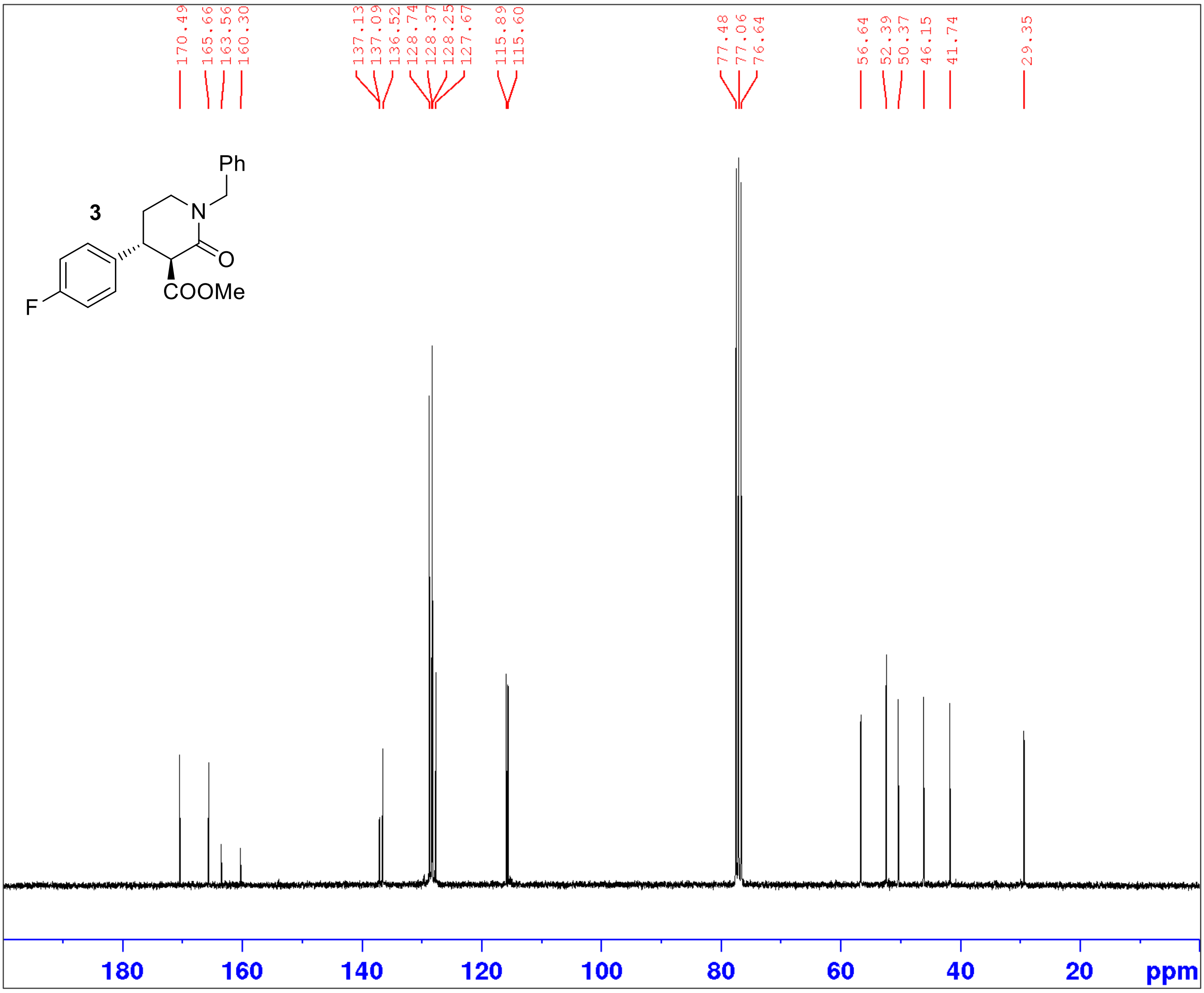

^{19}F-NMR (282 MHz, $CDCl_3$)

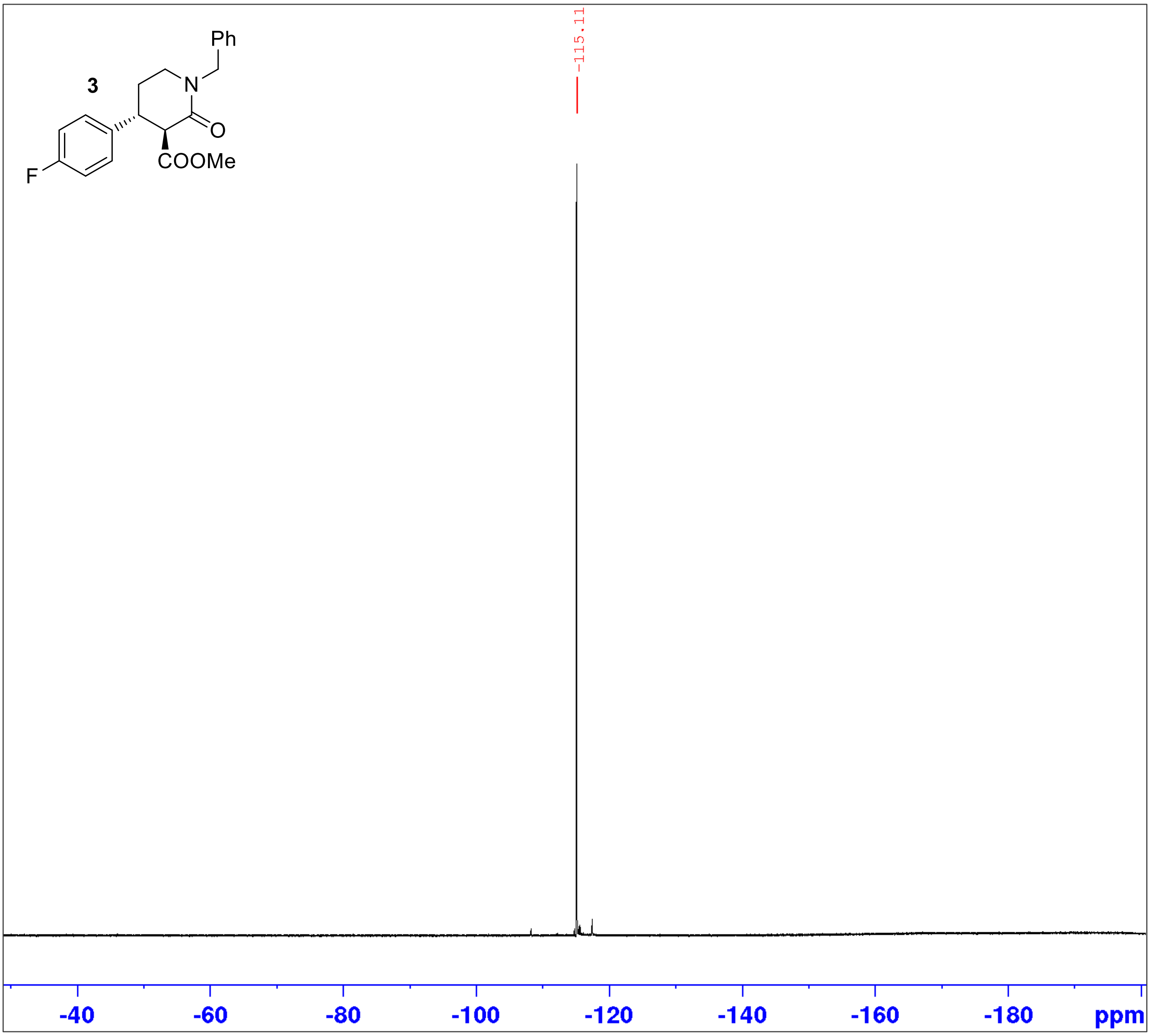

HPLC chromatograms

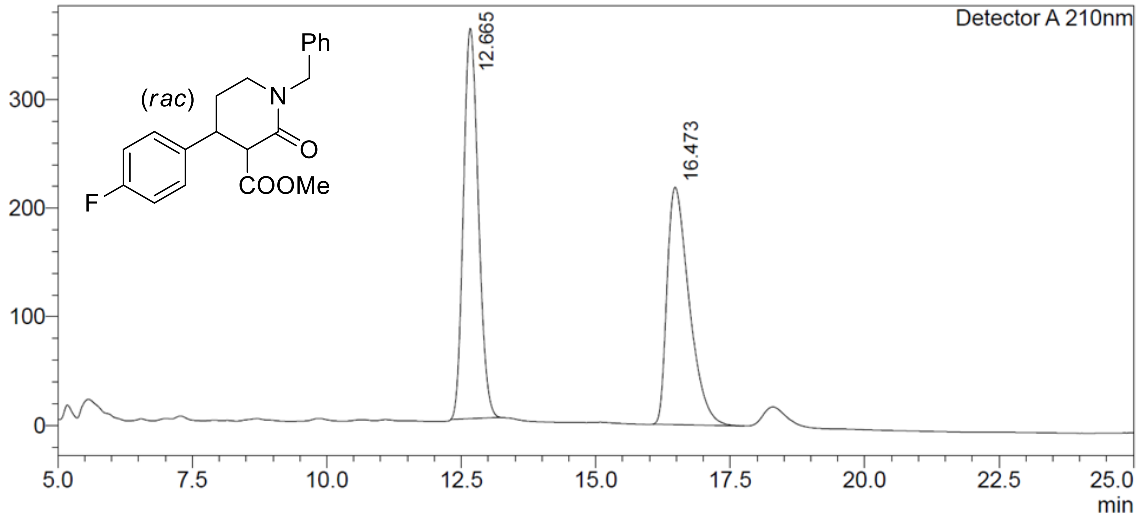

Peak#	Ret. Time	Area	Height	Conc.
1	12.665	6925109	359284	52.540
2	16.473	6255463	218548	47.460
Total		13180571	577832	

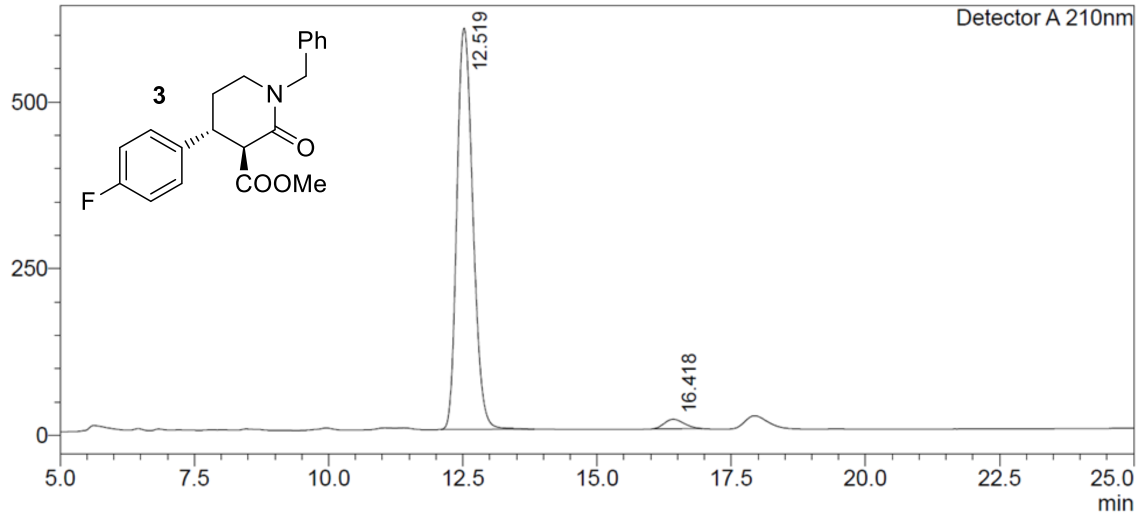

Peak#	Ret. Time	Area	Height	Conc.
1	12.519	12514096	601592	97.881
2	16.418	270900	12358	2.119
Total		12784996	613950	

The unassigned peek in HPLC chromatograms of **3** and (*rac*)-**3** at around 18 min belongs to the minor (*cis*) diasteroisomer.

((3*S*,4*R*)-1-Benzyl-4-(4-fluorophenyl)piperidin-3-yl)methanol (**1**)

^{1}H-NMR (300 MHz, $CDCl_3$)

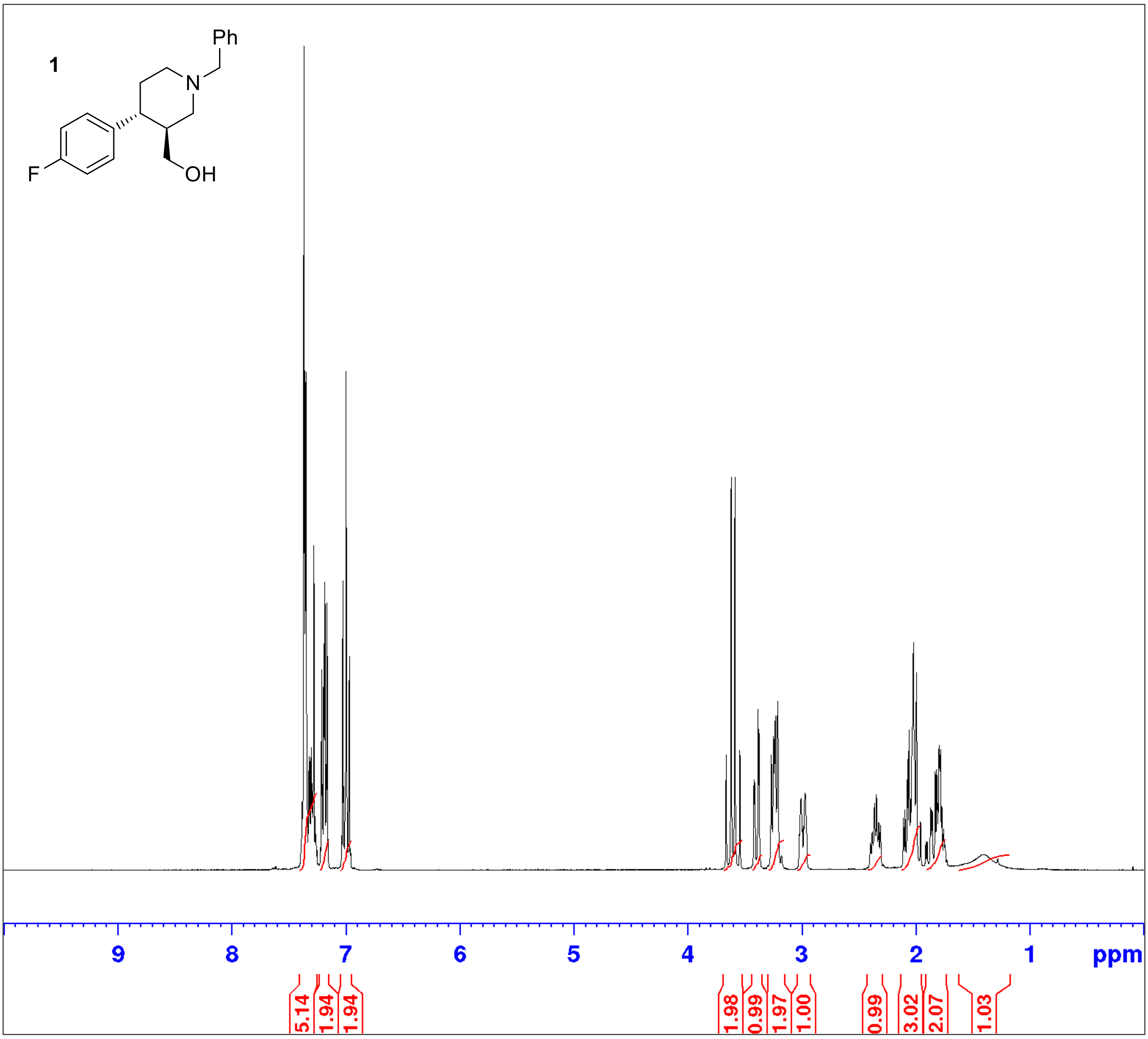

^{13}C-NMR (75 MHz, $CDCl_3$)

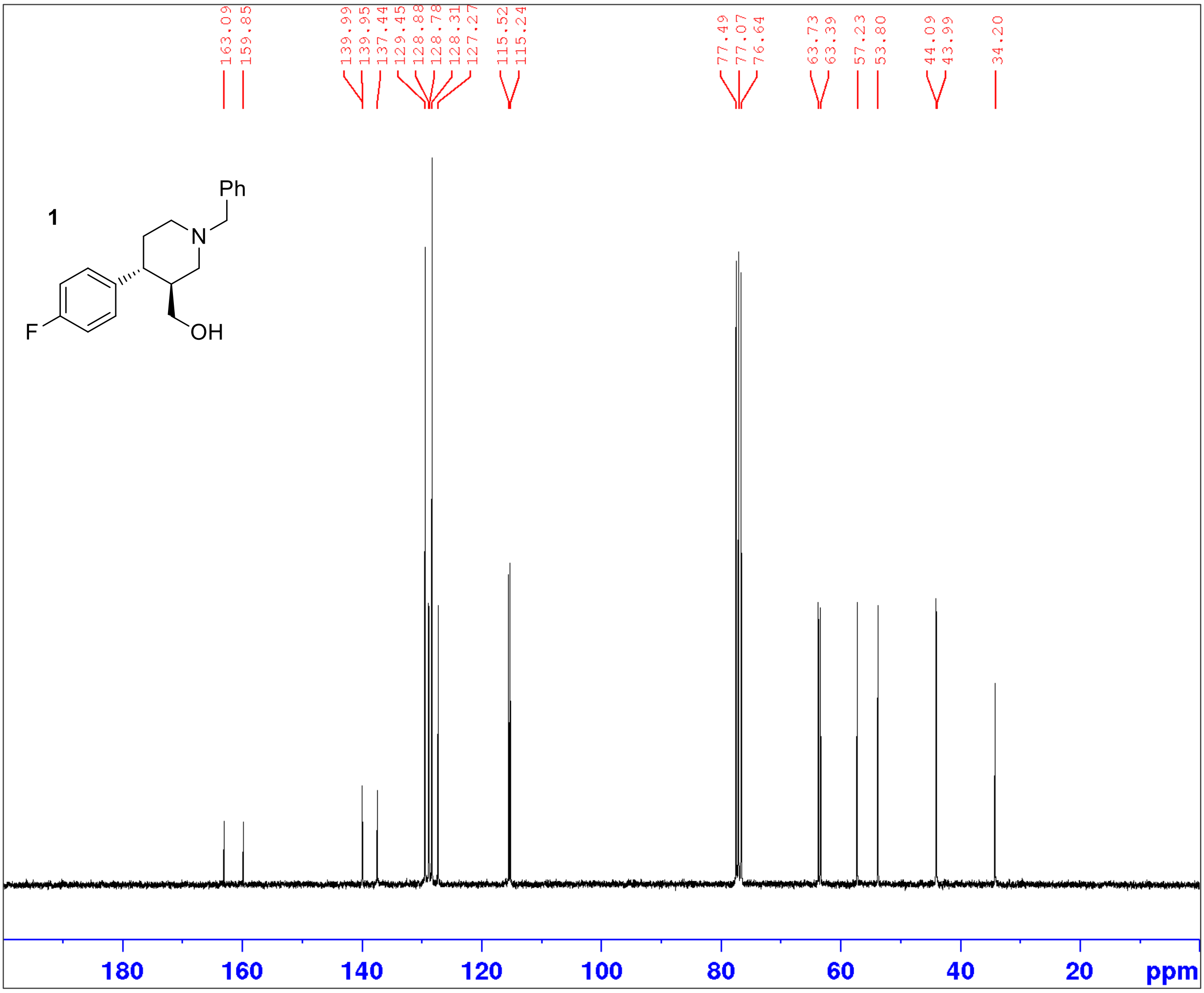

^{19}F-NMR (282 MHz, $CDCl_3$)

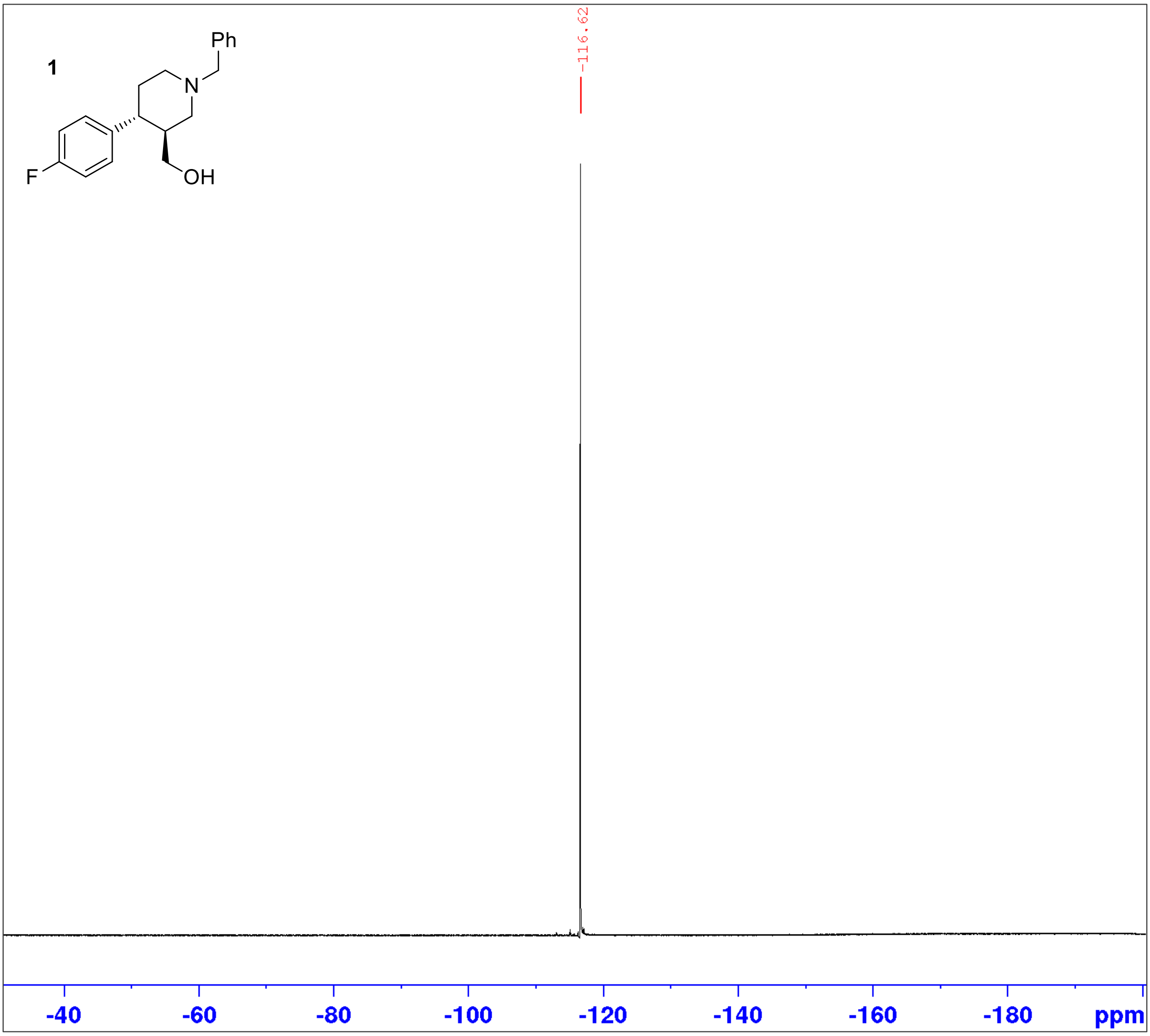

HPLC chromatograms

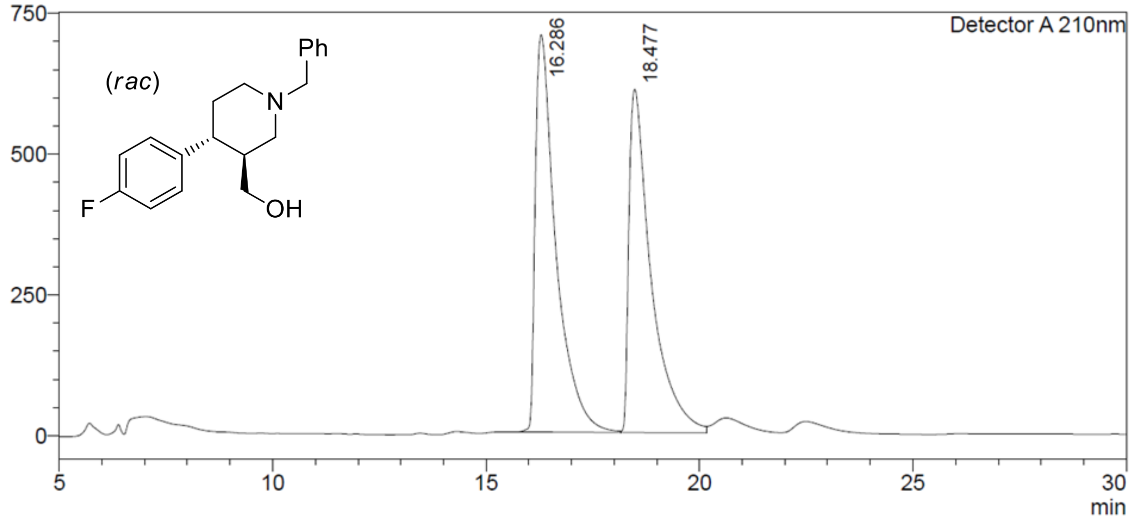

Peak#	Ret. Time	Area	Height	Conc.
1	16.286	23768107	705218	51.892
2	18.477	22035168	609507	48.108
Total		45803275	1314725	

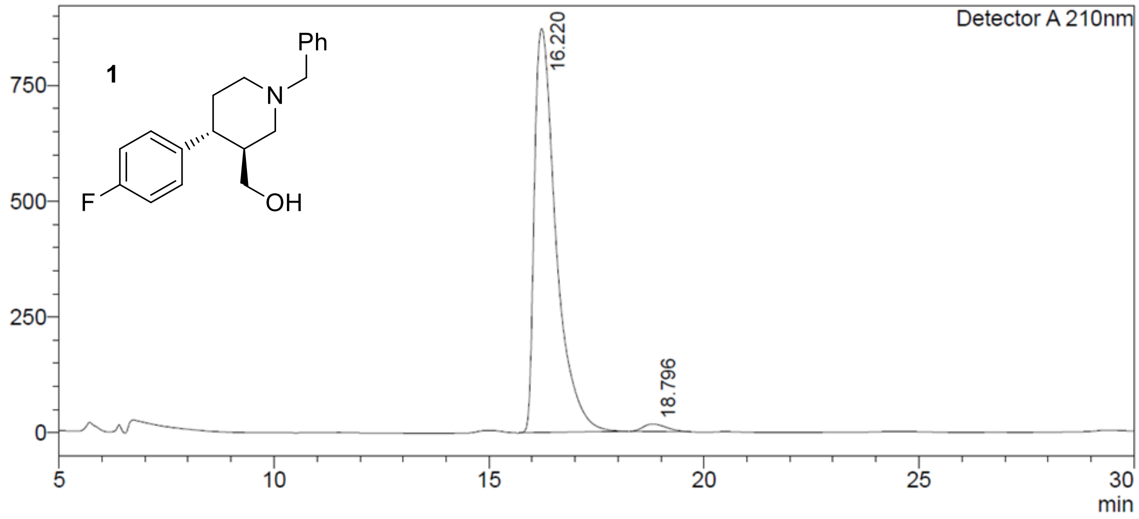

Peak#	Ret. Time	Area	Height	Conc.
1	16.220	30543833	873029	98.060
2	18.796	604242	16060	1.940
Total		31148075	889089	

9. References

(1) I. Arenas, A. Ferrali, C. Rodriguez-Escrich, F. Bravo, M. A. Pericas, *Adv. Synth. Catal.* **2017**, *359*, 2414–2424.

(2) J. Lai, S. Sayalero, A. Ferrali, L. Osorio-Planes, F. Bravo, C. Rodriguez-Escrich, M. A. Pericas, *Adv. Synth. Catal.* **2018**, 360, 2914–2924.

(3) J. Izquierdo, C. Ayats, A. H. Henseler, M. A. Pericas, *Org. Biomol. Chem.* **2015**, *13*, 4204–4209.

(4) A. Ma, S. Zhu, D. Ma, *Tetrahedron Lett.* **2008**, *49*, 3075–3077.

(5) J. F. Bower, T. Riis-Johannessen, P. Szeto, A. J. Whitehead, T. Gallagher, *Chem. Commun.* **2007**, 728–730.

(6) X. Wang, X. Wang, Z. Han, Z. Wang, K. Ding, *Angew. Chem. Int. Ed.* **2017**, *56*, 1116–1119.
